# Functional genomics of a generalist parasitic plant: Laser microdissection of host-parasite interface reveals host-specific patterns of parasite gene expression

**DOI:** 10.1186/1471-2229-13-9

**Published:** 2013-01-09

**Authors:** Loren A Honaas, Eric K Wafula, Zhenzhen Yang, Joshua P Der, Norman J Wickett, Naomi S Altman, Christopher G Taylor, John I Yoder, Michael P Timko, James H Westwood, Claude W dePamphilis

**Affiliations:** 1 Intercollege Graduate Program in Plant Biology, Huck Institutes of the Life Sciences, The Pennsylvania State University, University Park, Pennsylvania 16802, USA; 2 Department of Biology and Institute of Molecular Evolutionary Genetics, The Pennsylvania State University, University Park, Pennsylvania 16802, USA; 3 Department of Statistics and Huck Institutes of the Life Sciences, The Pennsylvania State University, University Park, Pennsylvania, 16802, USA; 4 Department of Plant Pathology, The Ohio State University, Ohio Agricultural Research and Development Center, Wooster, OH, 44691, USA; 5 Department of Plant Sciences, University of California, Davis, Davis, California, 95616, USA; 6 Department of Biology, University of Virginia, Charlottesville, VA, 22904, USA; 7 Department of Plant Pathology, Physiology and Weed Science, Virginia Polytechnic Institute and State University, Blacksburg, VA, 24061, USA; 8 Present address: Chicago Botanic Garden, Glencoe, IL, 60022, USA

**Keywords:** Parasitic plant, RNA-Seq, Illumina, *De novo* assembly, Transcriptome, Laser microdissection, Expansin, Generalist parasite, Orobanchaceae, *Triphysaria*, Maize, *Medicago*, *Burkholderia*

## Abstract

**Background:**

Orobanchaceae is the only plant family with members representing the full range of parasitic lifestyles plus a free-living lineage sister to all parasitic lineages, *Lindenbergia*. A generalist member of this family, and an important parasitic plant model, *Triphysaria versicolor* regularly feeds upon a wide range of host plants. Here, we compare *de novo* assembled transcriptomes generated from laser micro-dissected tissues at the host-parasite interface to uncover details of the largely uncharacterized interaction between parasitic plants and their hosts.

**Results:**

The interaction of *Triphysaria* with the distantly related hosts *Zea mays* and *Medicago truncatula* reveals dramatic host-specific gene expression patterns. Relative to above ground tissues, gene families are disproportionally represented at the interface including enrichment for transcription factors and genes of unknown function. Quantitative Real-Time PCR of a *T. versicolor* β-expansin shows strong differential (120x) upregulation in response to the monocot host *Z. mays*; a result that is concordant with our read count estimates. Pathogenesis-related proteins, other cell wall modifying enzymes, and orthologs of genes with unknown function (annotated as such in sequenced plant genomes) are among the parasite genes highly expressed by *T. versicolor* at the parasite-host interface.

**Conclusions:**

Laser capture microdissection makes it possible to sample the small region of cells at the epicenter of parasite host interactions. The results of our analysis suggest that *T. versicolor*’s generalist strategy involves a reliance on overlapping but distinct gene sets, depending upon the host plant it is parasitizing. The massive upregulation of a *T. versicolor* β-expansin is suggestive of a mechanism for parasite success on grass hosts. In this preliminary study of the interface transcriptomes, we have shown that *T. versicolor*, and the Orobanchaceae in general, provide excellent opportunities for the characterization of plant genes with unknown functions.

## Background

Approximately 1% of all extant angiosperm species are parasitic, deriving all or part of the water and nutrients from host plant species using specialized feeding structures known as haustoria. Among families containing parasites, only the Orobanchaceae contain species representing the full spectrum of parasitism from potentially free-living facultative forms to non-photosynthetic, obligate parasites [1]. *Lindenbergia* is a non-parasitic lineage of Orobanchaceae sister to all parasitic species [2]; therefore the family represents an ideal comparative framework to study the evolution of parasitism. Parasitic Orobanchaceae growing in Africa and the Mediterranean include the devastating agricultural pests witchweed (*Striga*) and broomrape (*Orobanche* and *Phelipanche*)*,* respectively. The *Striga* infestation covers 123.5 million acres resulting in annual yield losses greater than US$7 billion [3,4]. Broomrapes threaten nearly 40 million acres, though yield losses are difficult to assess due to the frequent abandonment of infested fields and unreliable data on yield loss [5]. *Striga* is one of the primary biotic constraints to agriculture in Sub-Saharan Africa and the affected areas are increasing in size [6]. The weedy, parasitic Orobanchaceae also threaten parts of Asia, Europe, and North America [7].

Motivated by the agronomic threat presented by some parasitic Orobanchaceae, *Triphysaria versicolor* has been developed as a model parasitic plant for the family. As a transformable [8] and tractable facultative generalist parasite, *T. versicolor* represents an excellent species to investigate the evolution of parasitism, haustorium development, plant-plant communication, host-parasite interactions, and many other facets of parasite biology [9,10]. To discover processes important in parasitic plant biology, we focused our analysis on the unifying anatomical feature of parasitic plants, the haustorium. This modified root structure is adapted to enable feeding on the host and is unique to parasitic plants, thus it is a focal point for interactions between the parasite and host [11].

Heide-Jorgensen and Kuijt [12,13] showed that the haustorium of *T. versicolor* contains many specialized cells including haustorial hairs, a xylem bridge between the host and parasite, and transfer-like cells adjacent to vessel elements at the host-parasite interface. Although histological evidence for xylem connectivity between the haustorium of *T. versicolor* and its host is well documented [12,13], there is no evidence for phloem connectivity. However, there is evidence that phloem-mobile virus particles move between host and parasite in the holoparasite *Phelipanche* (syn. *Orobanche*) *ramosa*[14] and phloem continuity has been observed in *Orobanche crenata*[15]*.* The mechanisms of transport between host phloem and parasite phloem likely vary in different parasites from direct phloem connections [15] to transport via apoplastic pathways. Bi-directional movement of small RNAs between host and parasite has been documented in *T*. *versicolor* attacking transgenic lettuce [16]. The anatomy of the haustorial interface cells and empirical evidence for bi-directional transport point to the host-parasite interface as an epicenter of host-parasite dialogue.

Intimate symbioses tend towards specialization (e.g. parasitism) [17]. A true generalist strategy, where a parasite routinely feeds on many distantly related host species, is relatively uncommon in parasitic organisms [18]. At face value, this is surprising, because a broad host range provides more feeding opportunities. Seedlings of most parasitic plants, for example, must contact and parasitize a suitable host plant soon after germination [10], and access to a wider range of potential host plants should *increase* the likelihood of survival, regardless of the specific plants growing nearby [19]. Although less common than host plant specialists, many parasitic plant families do contain generalists, including some or all parasitic members of Orobanchaceae, Lauraceae, Convolvulaceae, Krameriaceae, and most of the 18 families of Santalales (sandalwoods, mistletoes and their relatives [20]).

If mutations that increase specialized feeding strategies increase in frequency when specific host resources are predictable [21], then traits associated with maintenance of generalist abilities are likely to decrease in frequency. If a generalist strategy involves the evolution of a general-purpose suite of genes that are necessary and sufficient to successfully parasitize a wide range of hosts, then such a trend could lead to a long-term stable generalist strategy. Alternatively, if generalists maintain distinct sets of genes specific to different hosts, then the long-term maintenance of gene sets for attacking different hosts may be unlikely unless there is frequent reinforcement by a diverse range of hosts.

*Triphysaria* (Orobanchaceae) is a generalist parasite that feeds on a highly diverse collection of angiosperms in nature, including at least 30 species in 17 families of monocot and eudicot host plants [22]. We reasoned that sequencing transcriptomes from the haustorium of *T. versicolor* grown on distantly related hosts would maximize the potential to identify both shared and host-specific patterns of gene expression. The transcriptome datasets of *T. versicolor* provide a unique opportunity to leverage newly established genomic resources of the Parasitic Plant Genome Project (PPGP, [23]) with well developed functional protocols including parasite-host co-culture [9,24], haustorium induction assays [25], and parasite transformation [8,16,26]. By characterizing the molecular signature of host-parasite interactions, we stand to gain insight into the processes underway in a generalist parasite that facilitate a broad host range and learn about the molecular mechanisms that can facilitate the generalist parasite strategy.

Two substantial hurdles emerge when characterizing the transcriptomes of *T. versicolor* haustoria. The first is that gene expression profiles of specialized cells in the haustorium become diluted when harvesting even the tiny haustorium (1–2 mm diameter) of *T. versicolor.* The excellent histology and electron microscopy work by Heide-Jorgensen and Kuijt [12,13] revealed cells residing at the host parasite interface that had transfer cell-like morphology. The anatomy of these specialized cells includes dense cytoplasm, numerous small vacuoles, a highly invaginated cell membrane, and a labyrinthine cell wall (for a review see [27]). We hypothesized that the small collection of interface cells, including those with transfer-cell like morphology, facilitate the elusive molecular interaction between host and parasite, making them excellent candidates for transcriptome analysis. The second hurdle is that discovery of genes and subsequent gene expression analysis on a genome-wide scale is difficult without a sequenced and well-annotated genome, which is currently lacking for *T. versicolor*. Next Generation Sequencing (NGS) technologies have emerged as powerful tools for exploring new genomes because the cost per base is substantially lower than traditional dye-terminator or even pyro-sequencing (454) methods [28]. In the wake of the NGS revolution several tools for data analysis (for a review see [29]), including high performance *de novo* transcriptome assemblers like Trinity [30], have emerged to facilitate transcriptome analysis in uncharacterized model systems.

To overcome the limitations of reference independent transcriptome analysis of small numbers of difficult to harvest cells, we developed methods to sample parasite-host interface cells from *T. versicolor* grown on the distantly related and sequenced model hosts *Zea mays* (B73) (monocot) and *Medicago truncatula* (A17) (eudicot) via Laser Pressure Catapult Microdissection (LPCM). We extracted and then amplified exceedingly small RNA samples via T7-based linear amplification and then deeply sequenced each of the amplified parasite-host interface transcriptomes. We assembled millions of paired-end Illumina reads *de novo,* annotated each assembly and then estimated levels of gene expression via read mapping to the *de novo* assembled transcriptome. Using this approach, we identified genes that were part of a host-specific response as well as those that are part of a shared response of *T. versicolor* to the different hosts. We also verified the host-specific differential expression pattern of two *Triphysaria* expansin genes. Expansins are among the few genes known to be differentially regulated in haustoria [31,32]. Analysis of expansin genes allowed us to verify the differential gene expression pattern present in the interface sequence data, and demonstrate the first evidence that a β-expansin is highly upregulated in *T. versicolor* when grown on the *Z. mays* host. Our results suggest that the maintenance of a generalist feeding strategy in *Triphysaria* involves both generalized and specialized gene responses that help us understand *Triphysaria’s* generalist feeding abilities.

## Results

### Parasite host co-culture and microdissection of the *T. versicolor* haustorium

*T. versicolor* and hosts were germinated and grown axenically in separate culture plates. To begin co-culture, hosts were transferred to fresh plates and *T. versicolor* were added and placed in close proximity (~1 mm) to host roots. The attachment rate of *T. versicolor* to host roots was ~90% for *M. truncatula* and ~50% for *Z. mays.* This difference was likely due to the more rapid growth rate of *Z. mays* (compared to *M. truncatula*) coupled with the confined dimensions of the co-culture petri dish rather than differential parasite-host compatibility. Where host roots remained more or less stationary on the agar growth medium during early phases of co-culture, the attachment rates of *T. versicolor* were high (>90%) and equivalent between *Z. mays* and *M. truncatula.*

**Figure 1:**
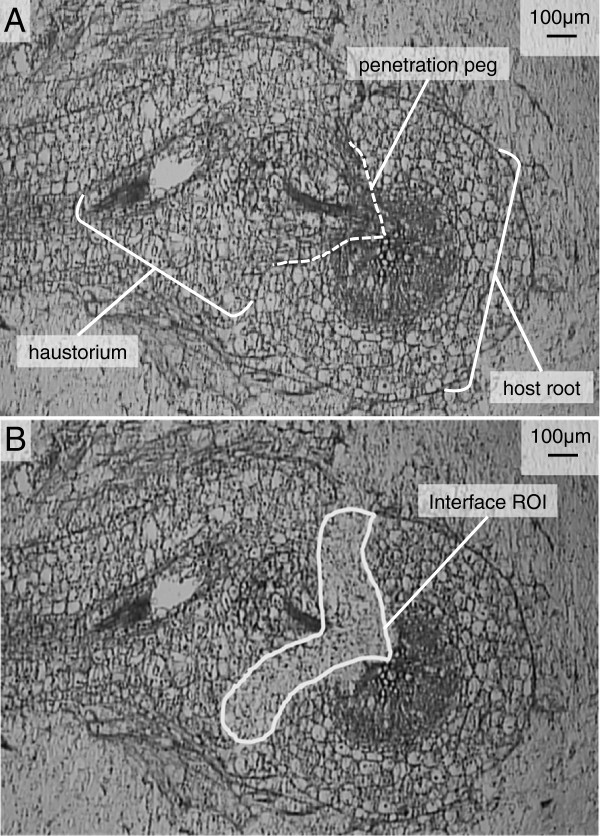
**Laser Microdissected Haustorium. **LPCM allows highly tissue- and cell-specific harvest after histological identification of tissues or cells of interest. **A**) Representative 25 μm cross-section of *T. versicolor* haustorium on the host *M. truncatula *approximately 9 days post infestation, and prior to LPCM. The mature haustorium contains the xylem bridge that connects the parasite and host vasculature and is visible in the penetration peg. **B**) The same section after LCPM shows the cleared interface tissue from the user-defined region of interest (ROI). The flakes of tissue are catapulted by a photonic cloud resulting from pulses of laser light focused between the tissue and glass slide. Multiple pulses of laser light raster across the ROI causing tissue in the selected region to be catapulted and then captured in the adhesive coated cap of a 0.5 mL tube held by a robotic arm in very close proximity (< 0.5 mm) to the upper surface of the section affixed to the slide.

The first step in sample preparation for LPCM was isolation and cryosectioning of haustoria formed on each host. The optimum section thickness was determined empirically by micro-dissecting samples from sections cut at 1μm section thickness intervals from 18-30 μm. For *T. versicolor* haustoria we determined that 25 μm cryosections were optimal to allow efficient tissue release from the adhesive coated StarFrost™ LPCM slides coupled with maximum tissue harvest volume. Parasite and host cells that were in contact with each other at the interface were difficult to separate, so to ensure capture of the entire parasite interface cell population we intentionally included a minimal amount of host tissue, knowing that host transcripts could be identified and removed informatically. Figure 1 shows a typical haustorium cross section before (A) and after (B) LPCM.

To generate representative interface-cell samples we pooled ~110 interface regions of interest (ROIs) from biological replicates (>8 haustoria). The average ROI for *T. versicolor* interface transcriptome samples grown on *M. truncatula* was 54,910 μm^2^ with a total area of 6.1 million μm^2^ that yielded 144 ng total RNA. This pooled sample had an RNA integrity number (RIN) of 7.6, an A_260_/A_280_ of 1.58 and an A_260_/A_230_ of 0.76. The average ROI for *T. versicolor* interface transcriptome samples grown on *Z. mays* was 56,079 μm^2^ with a total area of 6.4 million μm^2^ that yielded 160 ng total RNA with a RIN of 6.9, a A_260_/A_280_ of 1.68 and a A_260_/A_230_ of 0.11.

### Linear mRNA amplification from Laser Microdissected tissues

The first step of T7 based amplification is cDNA synthesis with an oligo-dT/T7 RNA primer/promoter. It is critical that this step is highly efficient to minimize the bias toward shorter fragment lengths in amplified samples [33]. We frequently observed a yellow-brown material at the host parasite interface that may have contributed to the low initial purity of the interface RNA samples, indicated by the low A_260_/A_280_ and A_260_/A_230_ ratios. Thus, we cleaned the interface total RNA with the Zymo™ RNA Clean and Concentrator kit. Subsequently, we observed consistent amplification performance between technical and biological replicates of the cleaned interface total RNA as well as performance consistent with positive control samples of *A. thaliana* young leaf RNA of high quality and purity (28s/18s ratio: 1.9; RIN: 8; A_260_/A_280_: 2.0, A_260_/A_230_: 2.1).

Amplification of ~100 ng of total RNA routinely yielded 50-100 ug of amplified RNA (aRNA) after two rounds of amplification, which was consistent with the positive control (*Arabidopsis* young leaf total RNA), the expected performance of the Message Amp™ II aRNA kit, and a previous report [34]. The aRNA yield after a single round of amplification was up to 100 ng, which is sufficient for construction of an Illumina sequencing library, yet we chose to amplify the samples for two rounds since it was desirable to have additional aRNA for further analyses including qRT-PCR validation of gene expression profiles. The fragment length profiles, as determined via Bioanalyzer™, were reduced from the first to the second round of amplification, which is consistent with a previous report [33].

### Sequencing and assembly statistics

Amplified interface RNA samples were sequenced on one lane each of Illumina’s Genome Analyzer IIx with an 83 × 83 bp paired-end cycle protocol. Sequencing data are available at http://ppgp.huck.psu.edu[23]. The *T. versicolor* interface transcriptome datasets (Table 1) contained 17.9 million read pairs on *Z. mays* and 19.1 million read pairs on *M. truncatula*. Host reads from each interface transcriptome dataset were mapped to their respective host genomes, leading to the removal of 1.5 million *M. truncatula* reads and 0.4 million *Z. mays* reads from each respective transcriptome data set. Reads were quality trimmed and filtered (see methods), leaving >26 million reads (orphans and mate pairs) for each sample that were then assembled separately using Inchworm (Trinity, [30]) and post-processed to remove exact duplicate or non-translatable sequences. The interface transcriptome assembly of *T. versicolor* grown on *Z. mays* yielded 12.77 Mbp of assembled sequence represented by 28,126 unigenes with an N50 of 525 bp (Table 1). The interface transcriptome assembly of *T. versicolor* grown on *M. truncatula* yielded 12.25 Mbp of assembled sequence represented by 26,709 unigenes with an N50 of 536 bp (Table 1). Sequencing and assembly statistics were similar in all categories (Table 1) indicating that both data sets were of equivalent quality.

**Table 1:** **Read and assembly level statistics for *T. versicolor *interface transcriptomes**

	**Host plant**	
**Sequencing**	***Z. mays***	***M. truncatula***
Total raw sequence	2.73 Gbp	2.91 Gbp
**Reads Total**	**35,894,662**	**38,228,134**
Host reads	(401,352)	(1,588,592)
Host filtered	35,493,310	36,639,542
Quality trimmed	26,947,737	27,325,845
**Assembly**		
Assembly length	12.77 Mbp	12.25 Mbp
**Unigenes Total**	**28,126**	**26,709**
Unigenes >500 bp	9,369	9,718
Min/Max length (bp)	197/3,115	197/3,265
N_50_	525 bp	536 bp
N_50 _>500 bp	731 bp	695 bp
**Annotation**		
Host unigenes	(4,967)	(7,785)
Non-plant unigenes	(127)	(329)
**Triphysaria unigenes**	**23,032**	**18,595**
Triphysaria hits	17,887	14,352
Other Plant hits	2,975	2,086
No hits	2,170	2,157

Low quality reads were filtered before assembly, and host sequences were filtered both before and after assembly. Unigenes remaining after removal of host plant and non-plant sequences were aligned with BLASTx to sequences detected in any other PPGP transcriptome library of *Triphysaria versicolor* (http://ppgp.huck.psu.edu/). Unigenes with less than 95% pairwise identity to either host or to other *Triphysaria* libraries were sorted further if a BLASTx search of the NR (http://www.ncbi.nlm.nih.gov) database yielded alignments of 1e-10 or stronger. The remaining unclassified unigenes were submitted to OrthoMCL DB and InterProScan. Unigenes that remained unclassified after the final screen are called “no hit” unigenes.

### Unigene annotation

Unigenes were annotated using an objective classification of known plant genes from the PlantTribes 2.0 database [35,36] as described in Wickett et al. [37]. We assigned genes into a hierarchy of gene clusters, which includes approximate gene families (Tribes), and potentially narrower lineages (Orthogroups) which seek to represent descendants of a single ancestral gene in the collection of reference plant species [35-37]. We also classified unigenes from our experiment using BLAST to query sequence databases (Table 1). To identify host derived unigenes in the mixed-species transcriptomes, we established a pairwise nucleotide identity threshold of 95% by querying a collection of *Z. mays* ESTs for *T. versicolor* grown on *M. truncatula* and *vice versa* (Additional file 1: Figure S1). The plot of *T. versicolor* unigene identity to each host database is clearly divergent at 95% compared to the reciprocal host database query. To verify that the incident high identity was not due to cross contamination between samples, we also queried the host EST databases with a *de novo* transcriptome assembly of *Lindenbergia philippensis,* a non-parasitic member of the Orobanchaceae [23]. The BLAST identity plot of the *Lindenbergia* transcriptome shows a similar trend to the plot of *Triphysaria* interface transcriptomes queried against the respective non-host databases (Additional file 1: Figure S1).

In order to remove host genes that may have escaped detection during pre-assembly read mapping, we further screened the assembly based on sequence similarity to cDNA (Phytozome, [38]) and EST databases (PlantGDB, [39]). This screen removed 4,967 unigenes from the transcriptome of *T. versicolor* grown on *Z. mays* and 7,785 unigenes from the transcriptome of *T. versicolor* grown on *M. truncatula* (Table 1)*.* The same reference transcripts used to screen the raw read data were also used to screen assemblies. The large number of putative host derived unigenes indicates that read screening with Mosaik at default values alone was insufficient to remove all host contamination. After the host screen the remaining unigenes were filtered for *T. versicolor* genes based on sequence similarity to genes detected in other PPGP libraries of *Triphysaria versicolor*[23]. We identified 17,887 unigenes from the transcriptome of *T. versicolor* grown on *Z. mays* and 14,352 unigenes from the transcriptome of *T. versicolor* grown on *M. truncatula* that had >95% identity at the nucleotide level to *T. versicolor* genes from the other PPGP libraries. After removing unigenes with high similarity to *T. versicolor* unigenes from other assemblies in the PPGP database, the remaining 5272 unigenes in the interface transcriptome of *T. versicolor* grown on *Z. mays* and 4572 unigenes in the interface transcriptome of *T. versicolor* grown on *M. truncatula* were used to query the non-redundant protein sequence database (NR) at NCBI [40] using BLASTx at a threshold e-value of 1e-10. Roughly half of the remaining unigenes in each interface transcriptome had best hits to plants including the model species *Arabidopsis, Populus*, and *Vitis,* other Orobanchaceae, or >30 other plant species (“Other Plant Hits” Table 1).

Each *T. versicolor* interface transcriptome had ~2300 unigenes with no significant alignments to sequences in any of the above described external databases (Table 1). We took several additional steps to try to identify these unknown sequences. Though these unigenes are not classified by source, we identified potential plant gene orthologs for ~20-25% of the remaining unigenes via the query of the PlantTribes 2.0 database. We then queried the extensive InterProScan (IPS) [41] and OrthoMCL DB [42] databases with the translated sequences of the remaining, unclassified unigenes. The majority of these unigenes (>75% in each transcriptome) lacked significant similarity to genes in the OrthoMCL database, nor did they contain IPS peptide motifs (Additional file 2: Figure S2); they are thus referred to as “no hit” unigenes (Table 1). The scan of OrthoMCL DB resulted in identification of an additional 100 plant and 7 non-plant unigenes from the *Zea* grown *T. versicolor* and 85 plant and 16 non-plant unigenes from the *Medicago* grown *T. versicolor* (Table 1)*.* Additionally, 370 *Zea* grown *T. versicolor* and 310 *Medicago* grown *T. versicolor* unigenes contained IPS motifs. Roughly half of the OrthoMCL DB hits lack descriptions or have minimal (e.g. one word) descriptions (Additional file 3). A similar pattern exists in the IPS search results, where about half of the unigenes with IPS motifs contain only putative secretion signals and/or transmembrane domains (Additional file 3). Overall our efforts to classify the unigenes in each assembly resulted in identification of potentially orthologous sequences for 82% of the *Zea* grown *T. versicolor* interface unigenes and 88% of the *Medicago* grown *T. versicolor* unigenes. We were able to assign a putative origin to >90% of unigenes in both transcriptomes and only 5% in each transcriptome remain unclassified. Of these, 493 unigenes from the *T. versicolor* grown on *Medicago* assembly and 536 unigenes from the *T. versicolor* grown on *Zea* assembly are longer than 300 bp and have read support.

The interface transcriptome of *T. versicolor* grown on *Z. mays* and *M. truncatula* contained a total of 127 and 329 unigenes, respectively, with best hits to non-plant species (Table 1). The non-plant component of each interface transcriptome included best hits to 16 taxa shared by both interface libraries. These included *Escherichia*, *Aspergillus, Clavispora*, *Burkholderia*, and others. Among this set, *Burkholderia* was the most highly represented taxon (>20 fold increase over any other species) in the non-plant component of both interface transcriptomes. These interface *Burkholderia* sequences were not detected in the reference transcriptome (TrVeBC1, sequence identity cutoff >90%).

The remaining “no hit” sequences, especially those >300 bp with read support, could represent unannotated host or parasite genes, uncharacterized associated symbionts, or incidental contamination. All remaining unigenes not assigned to a host plant or non-plant source in the NR database (23,032 for *T. versicolor* on *Z. mays* and 18,595 for *T. versicolor* on *M. truncatula*) are considered collectively as “putative *T. versicolor* derived unigenes.”

### Comparative interface transcriptome profiles of *T. versicolor*

**Figure 2:**
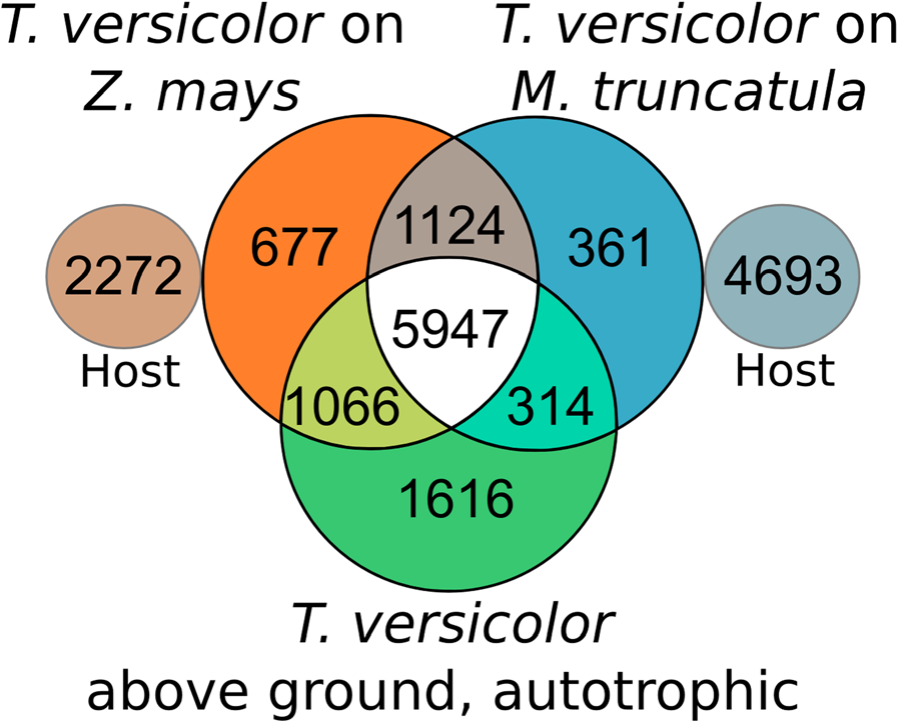
**Transcriptome Orthogroup Venn. **Venn diagram showing the number of Orthogroups in the interface transcriptomes of *T. versicolor *with hosts *Z. mays *and *M. truncatula *and an above ground, autotrophically grown *T. versicolor* transcriptome (TrVeBC1) constructed from leaves, stems and inflorescences. Also shown are the numbers of host-derived Orthogroups. The lack of overlap between host and parasite transcriptomes does not imply lack of shared Orthogroups, but indicates the total number of host Orthogroups for a point of comparison.

To examine the profiles of the *T. versicolor* interface transcriptomes, we used an annotated transcriptome from above ground tissues of autotrophically grown *T. versicolor* as a reference ([23]*Triphysaria* assembly TrVeBC1). We sorted unigenes by (PlantTribes 2.0) Orthogroups to identify host-specific and shared components of the interface transcriptomes of *T. versicolor* grown on *Z. mays* and *M. truncatula* (Figure 2)*.* As expected, the largest number of Orthogroups (5947, or 53.6% of the total detected in *T. versicolor*) were shared between all three transcriptomes, and likely represent expression of genes involved in processes common to a wide variety of cell types. A large number of Orthogroups (1124) were shared between the interface transcriptomes of *T. versicolor* interacting with both hosts. These genes likely include a putative core set of parasite genes that are active irrespective of the host plant species. Many additional Orthogroups were either exclusive to the interface and host-specific (677 for *Z. mays* and 361 for *M. truncatula*), or shared with above ground phases of growth (1066 for *Z. mays* and 314 for *M. truncatula*).

**Figure 3:**
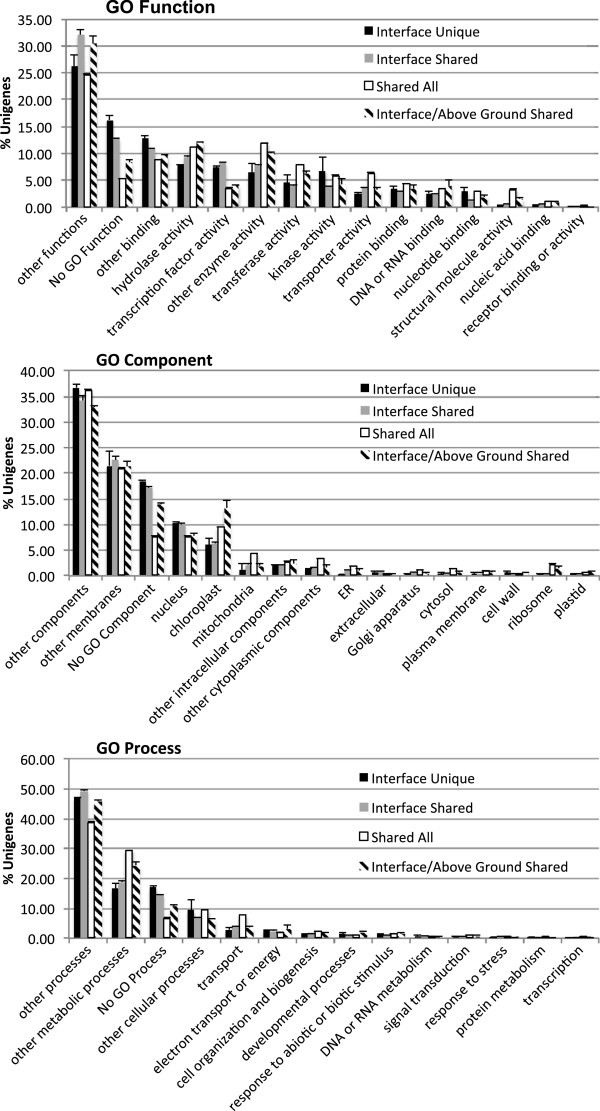
**GO Slim category summary. **GO Slim category terms of unigenes in interface transcriptomes of *T. versicolor *and the above ground reference assembly of *T. versicolor. *Each series displays the average number of unigenes in equivalent transcriptome components with a given GO Slim term. For instance, “Interface Unique” indicates the average number of unigenes from interface unique components in both *Medicago *and *Zea *grown *T. versicolor *transcriptomes. Error bars are standard error of the mean. “Interface Unique” = unigenes from Orthogroups that are host and interface specific, “Interface Shared” = unigenes from Orthogroups that are interface specific and shared between interface transcriptomes, “Shared All” = unigenes from Orthogroups shared between both interface transcriptomes and the above ground transcriptome, “Interface/Above Ground Shared” = unigenes from Orthogroups that are shared between the above ground, autotrophic transcriptome and the host-specific interface transcriptome.

Our annotation strategy includes assignment of a GO Slim category term derived from the best BLAST hit in PlantTribes 2.0. GO Slim categories are the broadest designations of GO and are useful for transcriptome-wide comparisons. The host-specific component of the *T. versicolor* interface transcriptome is likely to contain genes that interact with unique aspects of host biology while those that are shared likely contain genes essential for parasitism. To determine if the annotation profiles were similar between the overlapping and unique transcriptome components we plotted the proportion of GO Slim categories of unigenes (Figure 3) represented by unique or overlapping Orthogroups in Figure 2. GO Slim category profiles between equivalent components of each interface transcriptome were generally similar to each other, yet often distinct from profiles of non-equivalent components of each interface transcriptome (Figure 3). For instance, interface-unique Orthogroup profiles were similar between both interface transcriptomes, yet distinct from the above ground Orthogroup profiles of both interface transcriptomes.

GO Slim category profiles in overlapping and unique sets of Orthogroups within each interface transcriptome were tested for proportionality by a Chi-Square test (Additional file 4: Figure S3A-F). The results of all 6 tests showed disproportionality and were strongly significant (P<<0.0001). The number of unigenes in each GO Slim category, with strong residual values (strongly positive or strongly negative, thus disproportionate), is indicated in Additional file 4: Figures S3A-F. The results of this analysis are concordant with the plot of GO Slim category profiles (Figure 3). The most striking result is the consistently strong over-representation of unigenes lacking GO Slim categories in interface-unique Orthogroups (Additional file 4: Figure S3A-C: columns A and B, Figure S3D-F: columns E and F) compared to the consistent weak representation of unigenes lacking GO Slim categories in the shared Orthogroups (Additional file 4: S3A-C: column D, Figure S3D-F: column H). Interestingly the Orthogroups shared in the interaction of *T. versicolor* with both hosts are overrepresented by “transcription factor activity” GO Slim Function terms (Additional file 4: Figure S3A: column B and 3D: column F) and underrepresented by “transport” GO Slim Process terms (Additional file 4: Figure 3: column B and 3F: column F). This indicates that there are transcription factor genes active at the parasite-host interface that are not expressed in the above ground reference transcriptome. In contrast, the transporter gene families expressed at the interface are expressed in the above ground reference transcriptome as well.

### Highly expressed genes at the host-parasite interface

An advantage of using a *de novo* assembled transcriptome for RNA-Seq is an intrinsic threshold for transcript detection. If the transcript is represented by sufficient reads for *de novo* assembly, the presence of a target for *de novo* RNA-Seq is evidence for the presence of a transcript. The reference assembly TrVeBC2 [23] includes data from the haustorium of *T. versicolor* grown on *M. truncatula* and was used as a reference to map reads from each interface transcriptome. We correlated normalized reads (reads/kilobase/million mappable reads (RPKM)) from unigenes belonging to Orthogroups shared between the interface transcriptomes and the above ground reference transcriptome, TrVeBC1 (Additional file 5: Figure S4). For unigenes detected in both interface transcriptomes the correlation was high (Pearson’s R= 0.81), which indicates low technical and biological variability between the interface transcriptomes.

To determine the expression level of each unigene we also mapped reads to each respective interface *de novo* assembly. The 20 most highly expressed unigenes in each set of shared and unique Orthogroups from the two transcriptomes are presented in Additional file 6: Figures S5A-C. We queried this set of 120 unigenes against the NR database using BLASTx (e-value threshold: 1e-10) and 17 annotated plant genomes using BLASTn and BLASTx. The results of the database queries using BLAST are presented in Additional file 6: Figures S5A-C. The best-hit descriptions from searches in NR were concordant with the annotations assigned using PlantTribes 2.0 (See BLAST results in Additional files 7 and 8). We also used InterProScan to predict signal peptides and transmembrane domains for the unigenes listed in Additional file 6: Figure 5A-C (Additional files 9 and 10). The motif prediction tools frequently identified putative transmembrane domains in unigenes annotated as transporters and secretion signals in unigenes annotated as secretory proteins. Of the 120 unigenes listed (Additional file 6: Figures S5A-C), nine had no hit when queried against NR. The remaining unigenes had best hits to plant species. About 30% of these 120 unigenes have either no BLAST hits in NR or align to predicted, hypothetical, or otherwise uncharacterized sequences. This result is consistent our finding that the interface is enriched with unigenes that lack GO Slim category assignments (thus functional annotations).

Among the most highly expressed genes in the shared orthogroups of interface samples of *T. versicolor* grown on in both *Z. mays* and *M. truncatula* (Additional file 6: Figure S5A) are a β-expansin gene (see below), genes for several other cell wall modifying enzymes, and a gene encoding a putative ap2-erf domain transcription factor. A striking pattern in the shared interface Orthogroups was 10 unigenes (including six of the most strongly expressed unigenes from *T. versicolor* grown on *Medicago*) with sequence identity to annotated pathogenesis-related proteins in other eudicot species. A single *M. truncatula* unigene passed through the host plant removal process in the common interface component (ID 5537); this also shared high sequence identity with a pathogenesis-related protein.

Of the unigenes listed in Additional file 6: Figures S5A-C, 42 from the interface transcriptome of *T. versicolor* grown on *Z. mays* and 34 from the interface transcriptome of *T. versicolor* grown on *M. truncatula* had strongest BLAST hits to Asterid genomes (including *Mimulus guttatus*)*.* When we queried the 17 plant genomes database there were slightly more best hits to legumes in the *Medicago* grown *Triphysaria* data set, perhaps because there is less sequence divergence between the eudicots *Triphysaria* and *Medicago* than between the more distantly related *Triphysaria* and *Zea.* This results in a somewhat broader range of ambiguous sequence identity between host and parasite. Despite rigorous filtering, a single putative *Z. mays* transcript and three putative *M. truncatula* transcripts persevered (indicated in bold) in the highly expressed gene list in Additional file 6: Figure S5.

### A novel β-expansin is differentially expressed at the parasite-host interface

Among the highly expressed unigenes observed in the interface transcriptome of *T. versicolor* grown on *Z. mays* was a putative β-expansin (Additional file 6: Figure S5A). Manual curation of the read mapping data indicated that it was highly expressed when grown on *Z. mays*, and a nearly identical unigene from the *M. truncatula-*grown *Triphysaria* interface was lowly expressed. This apparently host-specific gene expression pattern was of interest because expansins are cell-wall loosening proteins (for a review, see [43]) that have been implicated in the interaction between parasitic plants and their hosts [31,32]. While the β-expansin gene was expressed in both samples, read mapping evidence suggested that this gene (unigene 772) was highly differentially expressed. As a point of comparison, we investigated a putative α-expansin, unigene 11, which showed a reciprocal pattern of high expression in the interface transcriptome of *T. versicolor* grown on *M. truncatula.* We verified the nucleotide sequence of unigenes 772 and 11 via dye-terminator sequencing of PCR products amplified from interface aRNA.

Phylogenetic analysis of β-expansin unigene 772 shows that it is nested within a supported clade of dicot β-expansin sequences (Additional file 11: Figure S6A) indicating that unigene 772 is a dicot β-expansin and not a *Z. mays* derived sequence. Annotation via InterProScan supports an expansin identity for 772 (Additional file 9) and shows a putative 5’ signal peptide (Additional file 6: Figure S5A), consistent with a role in the apoplast that is typical for expansins. Additionally, the results of all of the BLAST searches suggest that unigene 772 is a *T. versicolor* derived sequence. Phylogenetic evidence for unigene 11 does not yield a well-resolved tree of α-expansins (Additional file 11: Figure S6B), but the BLAST results suggest that the pairwise nucleotide identity to known, or putative (e.g. ESTs) *M. truncatula* genes is <70%, while unigene 11 has high identity (>95% pairwise nucleotide identity) to *Triphysaria* unigenes in other PPGP assemblies.

### Quantitative Real-Time PCR verification of host specific expansin expression

We sought to verify the reciprocal expression patterns of these two expansins via qRT-PCR. Unigenes 772 and 11 were assigned formal names TvEXPB1 and TvEXPA4, respectively. We verified that primers were specific to their targets by melting curve analysis. To further verify that the TvEXPB1, TvEXPA4, and TvActin primers were specific to parasite transcripts, we harvested portions of host roots that were immediately adjacent to mature *T. versicolor* attachments and interrogated them via qRT-PCR. In these host root samples we were able to detect ZmActin in *Z. mays* root samples and MtActin in *M. truncatula* samples, while the parasite primers yielded signal consistent with background.

**Figure 4:**
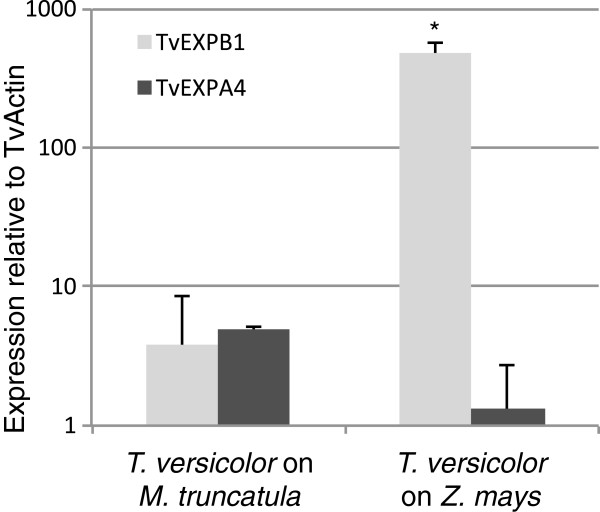
**Differential expansin expression. **qRT-PCR analysis of TvEXPA4 and TvEXPB1 expression relative to TvActin in parasite-host interface cells harvested by LPCM from the haustoria of *T. versicolor* *P<0.05.

We interrogated biological replicates of the *T. versicolor* host-parasite interface cells grown on both *Z. mays* and *M. truncatula* via qRT-PCR for expression of the reference gene, TvActin, TvEXPA4, and TvEXPB1 (Figure 4). TvEXPB1 is up-regulated >120 fold (P=0.024) in *T. versicolor* haustorial interface cells grown on *Z. mays* relative to *T. versicolor* grown on *M. truncatula.* TvEXPA4 shows a weak reciprocal pattern (P=0.17). The expression patterns of TvEXPA4 and TvEXPB1 are concordant with our *de novo* RNA-Seq results. Additionally, when we examined TvEXPB1 expression in whole haustorium samples the signal was indistinguishable from background, suggesting that the massive upregulation of TvEXBP1 is specific to a small number of interface cells.

## Discussion

Using a workflow that allowed us to sample, sequence, and *de novo* assemble transcriptomes from cells at the host-parasite interface we have shown that *T. versicolor* expresses genes in a host specific manner. This preliminary look at genes expressed at the parasitic plant-host plant interface suggests that the basis for generalist parasitism is constituted, at least in part, by host-specific patterns of gene expression. Generally, this work demonstrates the potential to discover genes *de novo* and examine genome-wide patterns of gene expression in a highly tissue-specific manner in organisms that lack a sequenced genome.

### Laser Microdissection (LM) is a powerful tool for plant transcriptomics

The power to develop a comprehensive picture of any biological system lies with understanding the myriad processes underway in complex organs and tissues. A primary hurdle to revealing this picture is the ability to identify, separate, capture and analyze tissues and cells of interest. Several authors have emphasized the importance of high resolution, high through-put investigations of gene expression in a tissue- and cell-specific manner as well as the need to survey gene expression in a global manner, and why LM (including LPCM) is emerging as a powerful tool for genomics [44-47].

LM generated samples from some model systems have been examined using microarrays [48-53] allowing investigators to analyze global gene expression patterns in specific tissues and cell types. More recently, LM has been increasingly coupled with NGS to sequence the transcriptomes of various tissues in *Z. mays*[54-56] and *S. lycopersicum*[34]. The advent of *de novo* transcriptome assembly now makes global surveys of gene expression in specific tissues and cells of non-sequenced organisms a logical next step. To examine the parasite-host interface transcriptome of *T. versicolor*, we combined LPCM with robust T7 based linear RNA amplification [33,57-60] in concert with Illumina mRNA-Seq, high performance *de novo* transcriptome assembly [30], and various assembly post-processing tools*.*

For the haustorium of *T. versicolor* our sampling strategy was based largely on detailed histology and transmission electron microscopy work done with field-collected specimens [12,13]. This critical background information allowed us to identify cells of interest within the haustorium and then subsequently identify regions of the haustorium in cryosections that contained these cells. Plant tissues must be embedded prior to LPCM and preparatory steps can have an impact on the quantity and quality of RNA preparation [61]. However, the ability to identify tissues of interest must be balanced with downstream usability. The histological quality of the section is an important consideration that may determine the sample preparation method. Paraffin embedded sections generally provide high histological quality at the expense of RNA quality and yield [62,63]. Histological quality increases with thinner sections for sampling at a finer spatial resolution and the efficiency of pressure catapulting increases with thinner sections, yet Kerk et al. [64] report increased RNA yield from thicker sections. We found that the optimum section thickness for capturing interface cells of the *T. versicolor* haustorium was 20-25 μm. This was determined based on a balance of our ability for histological identification of tissues and cells of interest with efficient tissue release from the slide during the pressure catapult phase of LPCM. The integrity of plant tissues that are susceptible to damage by flash freezing can be preserved by infusion with a cryoprotectant [65]. Cryosectioning with the CryoJane™ (a cryosection transfer system) allowed us to easily capture serial cryosections of *T. versicolor* haustorium that routinely yielded high quality RNA from carefully chosen samples.

By design, our sampling strategy minimized the likelihood that differences in gene expression arose from temporal or spatial sampling artifacts. The co-culture of *T. versicolor* is not highly synchronous, so our sample of haustoria represents a broad temporal window of connections that are ~8-10 days old. We collected ~110 interface ROIs which diminishes the likelihood of spatial sampling artifacts. Furthermore, highly similar statistics throughout sample processing and data analysis, including a high correlation of read counts for unigenes in shared Orthogroups (Additional file 5: Figure S4) and verification of the expression pattern of TvEXPB1 in additional experiments with biological replicates (Figure 4), indicates low variation from either biological or technical sources.

### Parasite-host interface transcriptomes help to identify core parasite genes

Orobanchaceae include the pernicious weeds *Striga*, *Orobanche* and *Phelipanche*, as well as the model parasite *Triphysaria.* We reasoned that by discovering a subset of genes that likely include those central to the response of *T. versicolor* to distantly related hosts, we would move closer to identifying a core set of parasitism genes operating in the weedy Orobanchaceae. The molecular dialogue of the parasitic plant with its host is largely uncharacterized [66]. Here we report the identification of the subset of genes expressed at the host-parasite interface by *T. versicolor* in response to both of the distantly related hosts *Z. mays* and *M. truncatula*. Additionally, we identified host-specific patterns of gene expression indicating that the generalist parasitic plant *T. versicolor* maintains suites of genes for use with different hosts.

The genes of *T. versicolor* expressed at the host-parasite interface in response to both *Z. mays* and *M. truncatula* included a substantial set of genes annotated in other plant species as pathogenesis- (or pathogen-response) related proteins. This included six of the most highly expressed genes in the *Triphysaria* interface grown on *M. truncatula*, and three when grown on *Z. mays*, but a number of additional unigenes were also putative homologs of genes that are upregulated during pathogen invasion (dirigent-like, acidic endochitinase, disease resistance proteins, etc.). While upregulation of genes of these classes would be expected as a defense response to pathogens, this observation suggests that pathways commonly involved in plant protection are also turned on by the parasite during the process of host invasion. It has been already suggested that parasitic plants may have recruited pathways from allelochemical detoxification for use during haustorium signal transduction [67]. Whether *Triphysaria* is defending itself during the invasion process with pathogen resistance (PR) genes, or has recruited PR pathways as offensive ‘weapons’ is not yet clear, but it does appear that a portion of the core gene set for *Triphysaria* parasitizing either host has been derived from pathways that plants use for defense against pathogens.

There is no consensus for why true generalist parasites occur or persist, even infrequently, despite evidence for an evolutionary trend toward specialization ([18] and references there-in). We hypothesized that a successful generalist feeding strategy might rely heavily on general-purpose genes that were deployed for feeding on any host. Instead, we see large components of the interface transcriptome that are detected only when feeding on one of the tested host plants. Of the 2162 Orthogroups detected only in interface transcriptomes, about half (52%) were expressed on both host species, but a substantial fraction of interface Orthogroups were detected only when grown on maize (31%), or on *Medicago* (17%). Long-term maintenance of extensive genetic machinery directed toward parasitism of subsets of possible hosts will require maintenance of selective pressures on the genes that are deployed in a host-specific manner. Unless the parasite routinely encounters and parasitizes a wide range of host plants, selective pressure on some host-specific genes will be relaxed and the gene functions eventually lost, limiting the plant’s potential to parasitize some hosts, despite the potential advantages of a large host range. The evidence for host specific gene expression suggests that *T. versicolor* regularly parasitizes across a broad host range, maintaining selective pressure on genes used in a host-specific manner. Indeed the advantage of a broad host range has been explored where host populations are moderately variable in space and time [19]. This study serves as a starting point for a broader survey of putative and potential hosts for *Triphysaria* as well as a first look at gene expression profiles of highly specialized tissues in parasitic plant haustoria.

It is intriguing that the host-specific and shared-interface gene families (Orthogroups) were over-represented by unigenes with no GO Slim category term assignment compared to the above ground transcriptome. Because Orthogroups were defined based on a classification of sequenced and annotated plant genomes [35-37] to which the parasite genes were assigned, this observation highlights the fact that genes expressed in the haustorium include many that have been recruited from the subset of genes whose function is not yet known in any plant. In addition, approximately 1300 unigenes from each interface transcriptome lack strong homology to any known sequence, though 25% of these unigenes are high identity reciprocal best hits in the interface data sets from each host. Further, because such patterns are reproducible in the interface transcriptomes of *T. versicolor* when grown on different host plants, these data suggest that genes of unknown function are expressed in the haustorium in a host-specific manner. Our data also suggest that underground phases of growth in *T. versicolor* are enriched for genes of unknown function.

Of those unigenes with GO Slim category assignments, the shared interface-specific Orthogroups are overrepresented for the GO Slim Function category “transcription factor” and underrepresented for the Go Slim Process category “transport.” This indicates that there are transcription factor Orthogroups unique to the interface (relative to the above ground reference transcriptome), yet Orthogroups involved in transport processes are active in all three transcriptomes examined. The latter observation regarding transport does not rule out differential expression of particular genes that are expressed in all three transcriptomes in this study.

### TvEXPB1 encodes a *T. versicolor* β-expansin that is part of a host-specific response

The expansin gene family includes 4 main groups: α, β, expansin-like A and expansin-like B [43]. Expansins are thought to loosen plant cell walls by allowing slippage of cell wall polymers [43]. Their activity is non-enzymatic, but they are distantly related to glycoside hydrolase family proteins [43]. Expansins are involved with cell growth [68] and have been implicated in the interaction of bacterial plant pathogens [69,70], plant-parasitic nematodes [71,72] and parasitic plants [31,32] with their plant hosts. In each each case, expansins are suggested to play a role in host invasion.

Expansin activity has been assayed in the cell walls of both monocots [73-75] and dicots [76,77]. β-expansins have activity that is specific to the cell walls of grasses, but not dicot or most other monocot cell walls [78]. The reverse pattern of action is found for α-expansins, which suggests substrate specificity for both α- and β-expansin proteins [43,79]. Typically, expansins are found at low concentrations [43] with the exception of grass pollen that secretes massive amounts of β-expansins [76] that likely serve to loosen stylar tissue during pollen tube growth [80,81]. Although the exact mechanism of action remains unknown, grass cell walls have relatively small amounts of xyloglucan and pectin; these are replaced with β-(1→3),(1→4)-D-glucan and glucuronoarabinoxylan. Both of these grass cell wall components are potential targets of β-expansins in their wall-loosening activity [82]. Throughout more than a decade of research, the accumulation of β-expansins to high levels was thought to be specific only to grass pollen [82].

In this study, we have shown that the transcript level for the β-expansin TvEXPB1 is among the most highly expressed genes in the interface transcriptome of *T. versicolor* grown on *Z. mays*. Relative to the interface transcriptome of *T. versicolor* grown on *M. truncatula,* the expression of TvEXPB1 is greatly up-regulated (>120-fold) in the parasite-host interface tissues of *T. versicolor* grown on *Z. mays.* The massive and host-specific expression of TvEXPB1 is suggestive of a role in a dicot parasite’s interaction with a grass host. Additionally, the signal for TvEXPB1 in whole haustorium samples of *T. versicolor* grown on *Z. mays* was undetectable relative to the interface samples suggesting that TvEXPB1 is highly specific to the host-parasite interface.

Taken together, the evidence for grass cell wall-specific activity of β-expansins and the massive upregulation of a parasite β-expansin at the parasite-host interface suggests that *T. versicolor* expresses TvEXPB1 when interacting with the monocot host *Z. mays* in an effort to manipulate host cell walls. Heide-Jørgensen and Kuijt [12,13] observed that host cortical and epidermal cells seemed crushed and displaced at the host parasite interface in the haustorium of *T. versicolor.* The mechanism of host tissue displacement may include cell wall modifying proteins like expansins that have host cell wall-specific activity. Such a mechanism would allow the parasite to soften or separate host cell walls without affecting the integrity of its own cell walls in the penetrating haustorium. The specific role that expansins play in the host parasite interaction will only be uncovered through detailed functional analysis. This includes focused gene expression analysis, targeted silencing of *T. versicolor* expansin genes and biochemical characterization to determine the substrate specificity of the expansin proteins encoded by these genes.

### Are a ubiquitous genus of soil bacteria symbionts of *T. versicolor?*

The best represented genus in the non-plant transcriptome component (~60% of non-plant hits in *T. versicolor* grown on *Z. mays* and ~45% non-plant hits in *T. versicolor* grown on *M. truncatula)* was *Burkholderia,* a common genus of soil bacteria. The high frequency of hits to this bacterium is surprising for three reasons: (1) the relative frequency of other non-plant genera was much lower, (2) *T. versicolor* seeds were aggressively surface sterilized prior to axenic co-culture, and (3) *Burkholderia-*derived unigenes were not detected in the above ground reference assembly*.* The low frequency of hits to other genera (including plant pathogenic fungi, human and other bacteria) could be explained by incidental contamination from the lab environment, however the preponderance of *Burkholderia* hits only in the interface samples suggest the presence of an organism belonging to this genus in the co-culture system. The unigenes were generally <1 kbp (indicating transcript-sized unigenes) and RNA samples were DNase treated, diminishing the likelihood that these unigenes originated from genomic DNA contamination.

The genus *Burkholderia* has received increasing attention in the last two decades due in part to a diverse catalog of host interactions ranging from human pathogen to plant-growth promoting rhizosphere fauna [83]. Of particular interest here is their potential role as beneficial plant endosymbionts [83]. Attsat [84] noted the presence of filamentous bacteria-like structures in haustorial cells and that application of terramycin significantly reduced haustorium formation in *Orthocarpus purpurascens* (syn. *Castilleja exserta*), a hemi-parasite that is a close relative of *T. versicolor*[85]. The presence of *Burkholderia* in an axenic co-culture system points to an intriguing possibility: a species of *Burkholderia* was carried through surface sterilization with the seeds of *T. versicolor.* Evidence that may suggest a role for a prokaryotic endosymbiont in parasitic plant biology [84] considered with evidence for the presence of a genus of beneficial soil bacteria hints at a symbiosis between *Burkholderia* and *Triphysaria.* The presence and possible roles of *Burkholderia* at the host-parasite interface in *T. versicolor* are currently under investigation.

## Conclusions

*Triphysaria* represents an important asset in the identification of genes and evolutionary processes that are central to parasitic plant biology, and one that is key to the development of new control strategies for the weedy Orobanchaceae. Highly tissue-specific transcriptome analysis in *Triphysaria versicolor,* an experimental model parasitic plant that has yet to have its genome sequenced, has revealed host-specific gene expression. Furthermore, genes represented at the parasite-host interface are enriched for genes of unknown function relative to above ground phases of growth. Ongoing development of molecular techniques, including parasitic-plant transformation [8,86] will facilitate the functional characterization of genes whose roles may be central in the parasitic plant-host plant interaction.

## Methods

### Growth of plant material

#### Triphysaria versicolor

Seed was collected from hundreds of open pollinated plants growing in coastal grassland stands of Napa California laboratories (University of California, Davis, USA) from the same source population as our prior transcriptome sequencing studies [37,87]. Seeds were surface sterilized in 70% ethanol for 10 min. while gently shaking, and then washed 3x with sterile distilled H_2_O. Seeds were further sterilized and scarified by a wash with a 50% bleach + 0.01% Triton X-100 (Sigma) solution for 30 min. while gently shaking. Seeds were then washed 10x with sterile distilled H_2_O and placed in petri dishes containing co-culture medium (1/4x Hoagland’s basal salt and nutrient mix, 7.5 g/L sucrose, 6 g/L plant tissue culture grade agarose, pH of 6.1) and wrapped with Parafilm™. Seeds were stratified for 4 days at 4°C in the dark then transferred to a 16°C growth chamber under a 12-hour light regime with a light intensity of 30 μmoles photons/m^2^/sec. *T. versicolor* seedlings were grown for 14–17 days then transferred to fresh co-culture plates with hosts.

#### Medicago truncatula (A17)

Seed was generously provided by Zengyu Wang (Noble Foundation, Oklahoma, USA). Seeds were scarified by incubation with occasional stirring in 18M H_2_SO_4_ for 8 min., and then washed 5× with sterile distilled H_2_O. Seeds were surface sterilized by a wash with 50% bleach + 0.01% Triton X-100 (Sigma) for 3 min. while gently shaking. Seeds were washed 10x with sterile distilled H_2_O and placed in a 50 mL conical bottom tube with 25 mL of sterile distilled H_2_O at 25°C, in the dark, over-night while gently shaking. The next day seedlings were transferred to sterile filter paper (Whatman #5), moistened with sterile distilled H_2_O, and placed in petri dishes. Seedlings were placed at 25°C, in the dark, over-night. Seedlings were transferred to co-culture medium and wrapped with Parafilm™ then placed in a growth chamber at 25°C under a 16-hour light regime with a light intensity of 100 μmoles photons/m^2^/sec for 5–7 days, then transferred to fresh co-culture plates with parasites.

#### Zea mays (B73)

Seed was generously provided by David Braun (University of Missouri, Columbia, USA). Seeds were surface sterilized by a wash with 20% bleach+0.08% Triton X-100 (Sigma) for 10 min. while gently shaking. Seeds were washed 3x with sterile distilled H_2_O. Seeds were further surface sterilized by a wash with 70% ethanol for 5 min. while gently shaking. The ethanol was decanted and the seeds were left to air dry in a laminar flow hood. Dry seeds were placed in petri dishes containing co-culture medium and wrapped with Parafilm™ then placed in a growth chamber at 25°C under a 16-hour light regime with a light intensity of 100 μmoles photons/m^2^/sec for 5–7 days, then transferred to fresh co-culture plates with parasites.

#### Parasite and host co-culture

*Zea mays* or *Medicago truncatula* were transferred to fresh co-culture medium at the times indicated above. Host roots were carefully oriented to allow placement of *T. versicolor* (grown as described above) seedlings in close proximity to host roots, with the parasite root tips 0.5-1 mm from the host root.Co-culture plates were sealed with Micropore™(3M) surgical tape and placed in a growth chamber at 25°C under a 16-hour light regime with a light intensity of 100 μmoles photons/m^2^/sec for 8–10 days.

### Tissue processing and sample preparation for sequencing

#### Tissue harvest

After 8–10 days co-culture, the haustoria of *T. versicolor* were harvested by making cuts in both the host root and parasite root ~1-2 mm adjacent to the haustorium under a stereomicroscope and were embedded in Shandon Cryomatrix™ (Thermo Scientific) dispensed in a 10 mm x 10 mm x 5 mm Cryomold™ (Tissue-Tek). Dissected tissue was quickly oriented with the host root on the vertical axis and with all haustoria equidistant from the bottom of the mold. Samples were quickly frozen on dry ice and stored at -80°C.

#### Cryosectioning and dehydration

Embedded haustoria were sectioned in a Cryotome SME (ThermoFisher Scientific) at 20-25 μm. Frozen sections were mounted to slides using the Cryo-Jane™ Tape Transfer System (Leica Microsystems). Mounted sections were dehydrated in a series of organic solvent baths (RT 70% ethanol (10 min.), 4°C 70% ethanol (2 min.), 4°C 95% ethanol (2 min.), 4°C 100% ethanol (2 min.), 4°C 100% xylene (2 min.), 4°C 100% xylene (2 min.), and finally RT 100% xylene (2 min.)). Slides were allowed to dry in a fume hood for 30 min.

#### Laser Pressure Catapult Micro-dissection (LPCM)

The P.A.L.M. Microbeam™ System (Zeiss) was used to harvest cells of interest. Only the pressure catapult function was used and the laser energy setting for the pressure catapult function was minimized. The laser focus was fixed, but the objective focus was manually adjusted to ensure efficient removal of tissues of interest during LPCM. Dissected tissue was captured in opaque adhesive cap 0.5 mL tubes (Zeiss, part #415101-4400-250) and stored at -80°C.

#### RNA extraction and RNA cleanup

Total RNA was extracted from LPCM harvested material using the PicoPure™ RNA isolation kit (Arcturus) with adaptations derived, in part, from documentation accompanying the opaque adhesive cap 0.5 mL tubes (Zeiss, part #415101-4400-250). The changes include modification of the RNA extraction step as detailed in the Arcturus protocol (Step 1) and are as follows: We removed the adhesive cap and placed it into the tube body of a 0.5 mL Eppendorf Safe-Lock™ tube (part # 022363719) to achieve a better tube-cap seal. 50 μL of buffer XB was added to the tube and it was vortexed inverted for 30 seconds. The tube was then placed in a temperature equilibrated (42°C) custom clamping device (not shown) to prevent leakage during the lysis step, in which the tube is inverted and incubated at 42°C for 30 min. in an air incubator. At 10 min. intervals the entire clamping device containing the tubes was vortexed inverted for 30 seconds. After the adapted lysis step we followed the PicoPure™ RNA isolation kit protocol beginning at step 2, RNA isolation. RNA was DNase treated on-column according to Appendix A in the PicoPure™ RNA isolation kit protocol. Total RNA was assessed on the Agilent Bioanalyzer using the RNA 6000 Nano kit (Agilent) with the Plant Total RNA assay with specific attention to the RNA Integrity Number (RIN, scale of 1 (degraded) to 10 (intact)) [88]. An additional clean-up of the total RNA prep was required to remove what we suspected to be poly-phenolics and secondary metabolites that interfered with downstream enzymatic treatments. The RNA Clean and Concentrator™-5 kit (Zymo Research) was used to clean and concentrate the total RNA extracted from LPCM harvested samples following the General Procedure in protocol version 2.0.6 and RNA was eluted in 10 μL RNase-free water.

#### T7 based RNA amplification of mRNA

The Message Amp™ II aRNA kit (Ambion) was used to amplify the poly-A RNA contained in total RNA samples to yield amplified RNA (aRNA). The input amount was approximately 100 ng of total RNA. The manufacturer’s instructions were followed and the first round of *in vitro* transcription (IVT) was allowed to progress for 14 hrs. The entire aRNA sample was concentrated in a Speed-vac (Savant) to <10 uls then entered into second round cDNA synthesis. The second round IVT was allowed to progress for 4 hours. Total RNA was assessed on the Agilent Bioanalyzer using the RNA 6000 Nano kit (Agilent) with the mRNA assay. Typical yields after the first round of amplification were up to 100 ng aRNA and yields after the second round of amplification ranged from 50-100 ug aRNA. High quality Arabidopsis young leaf RNA was used as a positive control and RT-PCR grade water (Ambion, included with the Message AmpII kit) was used as a negative control for amplification.

#### Illumina paired-end library construction

aRNA was used as input in Illumina’s mRNA Seq library prep protocol (Rev D). We omitted the poly-A selection step and moved directly to “Fragment the RNA.” 100 ng aRNA was fragmented and the manufacturer’s instructions were followed for the rest of the library preparation with the following exceptions: 1) we size selected the adapter-ligated library fragments at 300 bp rather than 200 bp at the “Purify the cDNA Templates” step, 2) we performed a second size selection/purification step by running a gel in a similar fashion as described in the “Purify the cDNA Templates” step and excised the band at approximately 325 bp that contained the products of library enrichment. The second size selection was done to purify the library and further constrain the fragment distribution as recommended by Illumina for paired-end mRNA Seq. The Illumina RNA Seq library was assessed on the Agilent Bioanalyzer using the DNA 1000 kit (Agilent).

### Sequencing, bioinformatics and phylogenetics

#### Sequencing

Paired-end (83×83 bp) sequencing was performed on the Illumina Genome Analyzer 2x by the Genomics Core facility at the University of Virginia in Charlottesville, VA USA. Each library was sequenced in one lane.

#### Post sequencing data processing and annotation

Contaminating sequences were removed from the pre-assembled, paired-end reads by alignment to the annotated coding DNA sequences of *Medicago truncatula*[38] and *Zea mays*[38] genomes using version 1.1.0014 of Mosaik Assembler [89] with the recommended parameters (hs = 15, mm = 12, and act = 35). Unaligned reads were then trimmed to remove low-quality bases (<Q20) from the ends using the quality trim program of CLC Assembly Cell version 3.2 (http://www.clcbio.com/index.php) requiring additionally that the remaining read fragment be at least half the original read length. Paired-end read files were reconstructed from the trimmed read fragments and orphaned single-end read fragments written to separate files using a custom script. The resultant filtered and trimmed set of reads for each library was then *de novo* assembled using the Inchworm component in release 03122011 of Trinity [30] with default parameters. Assemblies were filtered using version 2 of ESTScan [90] to remove sequences that had numerous frame-shift errors in coding regions, and version 4.0 of USEARCH [91] to remove similar (sub)sequences (to create non-redundant *de novo* assemblies). The post-processed unigenes for each build were then queried (BLASTx, 1e-5) against the 10 genomic proteomes in PlantTribes 2.0 [36,37] and assigned to PlantTribes 2.0 Orthogroups based on the cluster containing the best BLAST hit. Expression levels for each unigene was determined by mapping reads back to *de novo* assemblies (each interface transcriptome as well as TrVeBC1 and TrVeBC2) and computing RPKM value with the *High-throughput Sequencing RNA-Seq Analysis* program of CLC Genomics Workbench version 4.6 (parameters: mismatch cost = 2, insertion cost = 3, deletion cost = 3, length fraction = 0.5, similarity = 0.8, min insert size = 100, and max insert size = 250).

#### Post annotation assembly filtering

To determine the appropriate pairwise identity (at the nucleotide level) to reference host sequences, BLASTn was used to determine the frequency of unigene pairwise identity to reference host ESTs (*Zea mays* and *Medicago truncatula*, mRNA ESTs [39]). The interface transcriptomes of *T. versicolor* grown on *Z. mays* and *M. truncatula* were BLASTed into both the *Z. mays* and *M. truncatula* EST databases. The BLAST to the non-host databases was to determine the incident nucleotide pairwise identity of unigenes to a set of host references that should not be present in the assembly. To determine if unigenes with >95% identity to reciprocal host sequences were due to cross contamination, the distribution of unigene pairwise identity was determined for a whole-plant normalized assembly (of an non-parasitic member of the Orobanchaceae, *Lindenbergia philippensis* (PPGP assembly LiPhGnB1, [23]). An identity threshold of 95% was established to minimize host contamination while retaining parasite transcripts with incidentally high identity to the host reference sequences.

Final assemblies were filtered by BLASTn to remove unigenes with >95% identity to host derived sequences (*Zea mays* and *Medicago truncatula,* transcripts from [38], mRNA ESTs from [39]). Remaining unigenes were then filtered by BLASTn to available PPGP transcriptome assemblies of *T. versicolor*[23] to select for unigenes with >95% identity to other putative *Triphysaria* transcripts. Unigenes with less than 95% identity to host or *T. versicolor* sequences were queried against the non-redundant protein sequences database [40] using BLASTx (1e-10). Sequences were sorted by best-hit species into non-plant and plant categories. Sequences that remained unannotated and unclassified after extensive efforts detailed above were translated based on the ESTScan ORF prediction and submitted to InterProScan [41] via blast2go [92] using default parameters. These unigenes were also submitted to the OrthoMCL DB [42] database using default parameters.

The unigenes with the greatest RPKM in unique and overlapping Orthogroups, excluding those belonging to Orthogroups shared in all three transcriptomes and those unique to the above ground transcriptome, were used to query the non-redundant protein sequences database (NR) at NCBI [40] with BLASTx (1e-10) using blast2go [92]. This set of 120 unigenes was queried against a collection of 17 annotated plant genomes using BLASTn and BLASTx to determine best hits to annotated plant sequences not necessarily present in NR. The 17 genomes queried are as follows: *Arabidopsis thaliana, Carica papaya, Fragaria vesca, Glycine max, Medicago truncatula, Mimulus guttatus, Oryza sativa, Phoenix dactylifera, Populus trichocarpa, Selaginella moellendorffii, Solanum lycopersicum, Solanum tuberosum, Sorghum bicolor, Thellungiella parvula, Theobroma cacao, Vitis vinifera,* and. Additionally, these unigenes were annotated with InterProScan [41] via blast2go [92] with the default settings.

#### GO Slim category analysis

An annotated *T. versicolor* PPGP transcriptome with no tissue overlap of interface transcriptomes (TrVeBC1, [23]) was included in Orthogroup analysis to serve as a point of comparison for interface transcriptome analysis. Putative *T. versicolor* unigenes (excluding host and non-plant unigenes) were sorted based on Orthogroup assignment using Venny [93]. GO Slim annotations from unigenes present in Orthogroups were subject to a Chi-Square test using R [94]. Alpha was set to 0.05.

#### Phylogenetic analysis of TvEXPB1 and TvEXPA4

Homologs of TvEXPB1 from *Arabidopsis thaliana*, *Oryza sativa*, *Mimulus guttatus*, and *Selaginella moellendorfii* were extracted from Phytozome [38] v7.0: gene family #28891348 (β-expansins). Separately, alpha expansin homologs of TvEXPA4 were extracted from PlantTribes 2.0 [36] Orthogroup 1292. These sequences were combined with a subset of translatable sequences assembled in the PPGP project that had best BLAST hits with PlantTribes v2.0 Orthogroup 6163 (β-expansins) and 1292 (α-expansins). Inferred amino acid sequences for each data set were aligned using MUSCLE [95] and the coding DNA sequences were then forced onto this alignment for phylogenetic analysis. RAxML [96] version 7.2.8 was used to estimate the maximum likelihood tree under the GTR+gamma model of molecular evolution and bootstrap support values were estimated using 100 rapid bootstrapping replicates.

### Verification of Expansin transcript sequence and relative transcript abundance

#### cDNA synthesis

cDNA was synthesized from the same amplified samples used for sequencing, plus an additional biological replicate of each, to use as a template for qRT-PCR using the iScript^TM^ Reverse Transcription Supermix for RT-qPCR (BioRad). The manufacturer’s instructions were followed for all RNA samples as this kit utilizes both oligo-dT and random priming.

#### RNA isolation from host roots

RNA was isolated from host roots that were co-cultured, as described above, with *T. versicolor.* Host roots were harvested at sites adjacent to haustoria and flash frozen on liquid N_2_. Approximately 50 mg of tissue was vigorously macerated in Kimble Chase glass tissue grinders (part # KT885450-0020) in the presence of 450 μL buffer RLT + β-mercapto-ethanol (Qiagen) for 3 min. The lysate was then transferred to a QiaShredder column and the RNeasy Plant Mini Kit (Qiagen) instructions were then followed with the addition of an on-column DNase treatment (Appendix D: RNeasy Mini Handbook 4^th^ Edition) until elution, which was done in 30 μL RNase free water. Total RNA was assessed on the Agilent Bioanalyzer using the RNA 6000 Pico kit (Agilent) with the Plant Total RNA assay.

#### Primer design and sequence verification

Primers were designed using Geneious Pro (v5.5.4 [97]) to amplify TvEXPA4 and TvEXPB1 from aRNA based upon the unigenes that resulted from the *de novo* assembly and post-processing of the Illumina paired-end mRNA Seq data.

Primer sequences:

TvEXPA_F5: GCTTTTGCCTACGACCAACTTATG

TvEXPA_R3: GACAGTTTTGCCATCGCTTGTAG

TvEXPB_F1: GCCATAGTTTCAACCCGAGGAC

TvEXPB_R2: GGCTTCTTCCTGCTCTCCTTACTTG

With these primers the putative α- and β-expansins transcripts were amplified and submitted for Sanger sequencing. Sequencing reads were quality trimmed manually by visually examining the electroferrograms. MUSCLE was used to align the Sanger reads with the unigenes to confirm the unigene sequence. Any remaining gaps were sequenced with the same method using primers based on the Sanger sequence verified first-round PCR products.

#### Quantitative Real-Time Polymerase Chain Reaction

**(qRT-PCR)** To verify the host-specific expression pattern of TvEXPB1 and TvEXPA4 observed in the *de novo* read mapping results, transcript levels of TvEXPB1, TvEXPA4, TvActin, ZmActin, and MtActin were estimated using qRT-PCR.

Primer sequences:

TvEXPA4:

For: 5’-TGGGAGGTGCTTGTGGGTAT-3’;    Rev: 5’-CCGCAGGATAACCCATTGTT-3’

TvEXPB1:

For: 5’-GATGGCCTGACTGAAGTTGCA-3’;    Rev: 5’-GCGGCAAATTCACCCTAAAA-3’

TvActin:

For: 5’-ACCCGATCCTTCTCACTGA-3’;    Rev: 5’-CATGACAATACCAGTCGTACG-3’

ZmActinB:

For: 5’-CAATGGCACTGGAATGGT-3’;    Rev: 5’-ATCTTCAGGCGAAACACG-3’ [98]

MtActin:

For: 5’-ATGTTGCTATTCAGGCCG-3’;    Rev: 5’-GCTCATAGTCAAGGGCAAT-3’ [99]

#### Verification of primer specificity

To determine if the primers were specific to their intended targets, melt-curve analysis was performed for each PCR product. Primer specificity for parasite target genes was verified by submitting host RNA extracted from co-cultured host roots to analysis by qRT-PCR with parasite gene primers. In both cases host actin transcripts were detected, yet primer pairs specific to the parasite genes yielded signal consistent with background. All no-template controls (NTC) showed signal consistent with background signal and all reverse transcription negative (RTN) controls showed signal consistent with background.

#### qRT-PCR assay conditions

The qRT-PCR reaction prepared using the KAPA™ SYBR FAST qPCR kit (KAPA Biosystems, KK4602) following the manufacturer’s instructions. The reaction was run on a BioRad MyiQ (170–9770) with the following program:

95°C for 8 min. (initial melt)

95°C for 0.5 min. (cycle melt)

60°C for 0.5 min. (cycle anneal/extend)

Repeat 40 cycles

Melt Curve: 0.5°C increments from 95°C – 25°C.

#### qRT-PCR data analysis

Crossing point (Ct) values for each of 3 technical replicates were used to calculate the average Ct value. The 2^(-ddCt) method was used to calculate the fold-change in expression in each sample relative to the control [100]. A one-tailed, two-sample *t*-test assuming unequal variance was performed using R [94]; alpha was set to 0.05.

## Abbreviations

(NGS): Next Generation Sequencing; (LPCM): Laser Pressure Catapult Microdissection; (ROI): Regions of Interest; (RIN): RNA Integrity Number; (aRNA): amplified RNA; (T. versicolor): *Triphysaria versicolor*; (Z. mays): *Zea mays*; (M. truncatula): *Medicago truncatula*; (RPKM): Reads/kilobase/million mappable reads; (LM): Laser Micro-dissection; (PR): Pathogen resistance; (qRT-PCR): Quantitative Real-Time PCR; (NCBI): National Center for Biotechnology Information; (NR): Non-redundant protein sequences database.

## Competing interests

The authors declare that they have no competing interests.

## Authors’ contributions

Conception and design of PPGP transcriptome (JHW,,CWD, MPT, JIY) and interface transcriptome study (LAH, CWD); seeds and *Triphysaria* growth and assays: JIY; plant cultivation and tissues: LAH; laser capture methods and sampling: LAH, CGT; RNAs, amplifications, and sequencing libraries: LAH; qRT-PCR: LAH, ZY; data analysis and presentation: LAH, JPD, EKW, NJW, ZY, NSA, CWD; Wrote manuscript: LAH and CWD with contributions from all of the authors. All authors read and approved the final manuscript.

## Supplementary Material

Additional file 1: Figure S1 **Unigene Pairwise Nucleotide Identity Plot. **Sequence identity between unigenes considered in this study and reference EST sets (PlantGDB public ESTs, http://www.plantgdb.org/) for the hosts *Z. mays *and *M. truncatula. Triphysaria *unigenes were aligned to the host reference to identify host contaminants and aligned to the reciprocal non-host reference sets to identify the incidental nucleotide pairwise identity. A whole plant normalized transcriptome assembly of *Lindenbergia philippensis *(a non-parasitic member of the Orobanchaceae) was used to determine the distribution of pairwise identity for a non-parasite to each host and to control for high unigene identity to host ESTs from potential cross contamination. A threshold of 95% was chosen to balance exclusion of host transcripts with retention of *Triphysaria *unigenes that had incident high identity to host ESTs. Click here for file

Additional file 2: Figure S2 **VENN diagram summary of OrthoMCL DB and InterProScan (IPS) results. **ESTScan ORF predictions from unigenes in each interface transcriptome that remained unclassified after extensive BLAST-based database searching were translated and submitted to OrthoMCL DB and InterProScan. The pattern is similar between unigenes from each transcriptome indicating equivalent unigene classification for *T. versicolor *grown on both hosts. The number of unigenes for which an ortholog or peptide motif was identified was relatively small, indicating our unigene classification using PlantTribes 2.0 and external database queries was robust. Approximately 25% of the known orthologs identified in the OrthoMCL database from each transcriptome are shared.A majority of the unigenes remain unknown, and these include many (~500 in each transcriptome) that are >300 nucleotide bp and have read support. Click here for file

Additional file 3 OrthoMCL DB and InterProScan annotation summary spreadsheet of unigenes that remained after the screen of PPGP databases, host cDNA and EST sequences, and NCBI’s non-redundant protein sequences database. Click here for file

Additional file 4: Figure S3 **GO Slim category analysis. **Chi-Square test (P<<0.0001) of GO Slim terms represented in the indicated regions of the Venn. The numbers of unigenes in each GO category for indicated regions are listed in the table. Cells with strongly positive residual values (>4) are indicated as **bold+ **and strongly negative residual values (<-4) are indicated as **bold-**. GO Slim Function (**A**), Component (**B**) and Process (**C**) category analysis for the interface transcriptome of *T. versicolor *grown on *Z. mays*. GO Slim Function (**D**), Component (**E**) and Process (**F**) category analysis for the interface transcriptome of *T. versicolor *grown on *M. truncatula*. Click here for file

Additional file 5: Figure S4 **Correlation of normalized read counts (RPKM) for unigenes in orthogroups shared between the interface transcriptomes and reference assembly TrVeBC1 (ppgp.huck.psu.edu).** Reads from each interface transcriptome were mapped to a reference assembly (TrVeBC2, ppgp.huck.psu.edu) that included whole haustorium data from *T. versicolor* grown on *M. truncatula*. A subset of unigenes is more highly expressed in the interface transcriptome of *T. versicolor *grown on *M. truncatula*; a similar pattern is not observed for *T. versicolor *grown on *Z. mays*. This is due to a bias for *Medicago *grown *Triphysaria *unigenes in the reference dataset TrVeBC2, which was constructed with reads from *Medicago* grown *Triphysaria*. For unigenes in shared orthogroups, the RPKM values are highly correlated (Pearson’s R = 0.81) between interface transcriptomes indicating that technical and biological variation is low. Click here for file

Additional file 6: Figure S5 **Highly Expressed Interface Unigenes. **The 20 most highly expressed (RPKM) unigenes (ID) in each indicated portion of the transcriptome Venn diagram for the interaction of *T. versicolor *with each host species. NR BLASTx – description, species and %id.: the description, species of origin, and percent pairwise identify, respectively, of the best unigene alignment (<1e-10) resulting from the NR database query, 17 genomes BLAST and %id.: best hit species in a BLAST database of 17 annotated plant genomes with the percent pairwise identity in the nucleotide BLAST (N) or translated nucleotide BLAST (P). ^T^XXX = IPS transmembrane prediction, ^S^XXX = IPS secretion signal prediction. Click here for file

Additional file 7 **Text file (BLAST default output format) of BLAST results from the query of NCBI’s non-redundant protein sequences database with *Z. mays *grown *T. versicolor *unigenes listed in Additional file 6.** Click here for file

Additional file 8 **Text file (BLAST default output format) of BLAST results from the query of NCBI’s non-redundant protein sequences database with *M. truncatula *grown *T. versicolor *unigenes listed in Additional file 6.** Click here for file

Additional file 9 **Results from the InterProScan analysis of *Z. mays *grown *T. versicolor *unigenes listed in Additional file 6.** Click here for file

Additional file 10 **Results from the InterProScan analysis of *M. truncatula* grown *T. versicolor *unigenes listed in Additional file 6.** Click here for file

Additional file 11: Figure S6 **RaxML analysis of A: *Triphysaria *beta expansin gene TvEXPB1 (TrVeIntZeamaGB1_772, green text), and B: alpha expansin gene TvEXPA4 (TrVeIntMedtrGB1_11, green text). **Bootstrap proportions are given above each node. **Taxon abbreviations for A: ***Arabidopsis thaliana *(AT), *Oryza sativa *(Os), *Mimulus guttatus* (Mg), *Triphysaria versicolor *(TrVe), *Striga hermonthica *(StHe), *Phelipanche *(=*Orobanche*) *aegyptiaca *(OrAe), *Selaginella moellendorffii *(Smoellendorffii). **Taxon Abbreviations for B:***Oryza sativa *(Os), *Sorghum bicolor *(Sb), *Striga hermonthica* (StHe), *Phelipanche *(=*Orobanche*) *aegyptiaca *(OrAe), *Triphysaria versicolor *(TrVe), *Carica papaya *(Carpa), *Populus trichocarpa *(Poptr), *Medicago truncatula *(Medtr), *Vitis vinifera* (Vitvi), *Arabidopsis thaliana *(AT), *Selaginella moellendorffii* (Selmo), *Physcomitrella patens *(Phypa). Click here for file
