# Supplementary material for: Functional genomics of a generalist parasitic plant: Laser microdissection of host-parasite interface reveals host-specific patterns of parasite gene expression
Source: BMC Plant Biol. 2013 Jan 9;13:9. doi: 10.1186/1471-2229-13-9 (PMC3636017; doi:10.1186/1471-2229-13-9)

Supplemental Figure 1. Unigene Pairwise Nucleotide Identity Plot. Sequence identity between unigenes considered in this study and reference EST sets (PlantGDB public ESTs, http://www.plantgdb.org/) for the hosts *Z. mays* and *M. truncatula*. *Triphysaria* unigenes were aligned to host reference to identify host contaminants and aligned to the reciprocal non-host reference sets to identify the incident nucleotide pairwise identity. A whole plant normalized transcriptome assembly of *Lindenbergia phillepensis* (a non-parasitic member of the Orobanchaceae) was used to determine the distribution of pairwise identity for a non-parasite to each host and to control for high unigene identity to host ESTs from potential cross contamination. A threshold of 95% was chosen to balance exclusion of host transcripts with retention of *Triphysaria* unigenes that had incident high identity to host ESTs.

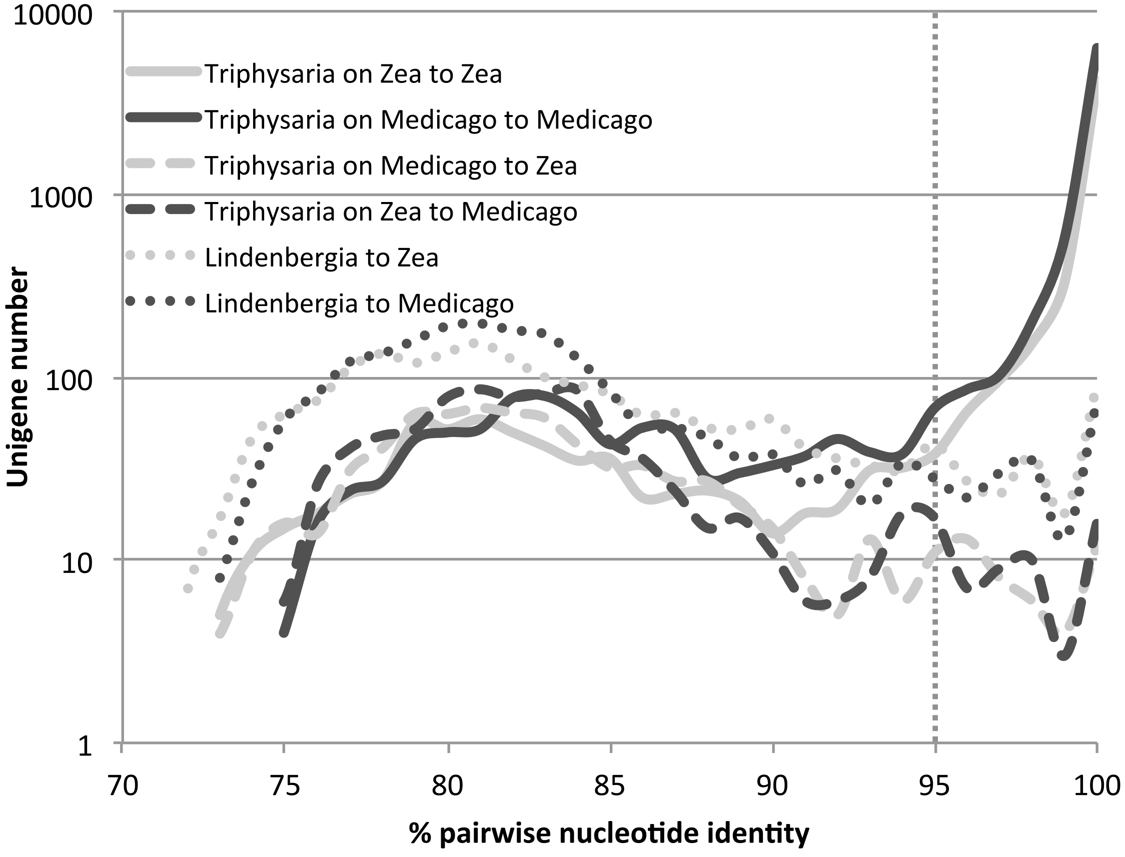

Supplement: Additional file 1: Figure S1 — Unigene Pairwise Nucleotide Identity Plot. Sequence identity between unigenes considered in this study and reference EST sets (PlantGDB public ESTs, http://www.plantgdb.org/) for the hosts Z. mays and M. truncatula. Triphysaria unigenes were aligned to the host reference to identify host contaminants and aligned to the reciprocal non-host reference sets to identify the incidental nucleotide pairwise identity. A whole plant normalized transcriptome assembly of Lindenbergia philippensis (a non-parasitic member of the Orobanchaceae) was used to determine the distribution of pairwise identity for a non-parasite to each host and to control for high unigene identity to host ESTs from potential cross contamination. A threshold of 95% was chosen to balance exclusion of host transcripts with retention of Triphysaria unigenes that had incident high identity to host ESTs. [file 1471-2229-13-9-S1.pdf]
