# Supplementary material for: Functional genomics of a generalist parasitic plant: Laser microdissection of host-parasite interface reveals host-specific patterns of parasite gene expression
Source: BMC Plant Biol. 2013 Jan 9;13:9. doi: 10.1186/1471-2229-13-9 (PMC3636017; doi:10.1186/1471-2229-13-9)

Supplemental Figure 2. VENN diagram summary of OrthoMCL and InterProScan (IPS) results. ESTScan ORF predictions from unigenes in each interface transcriptome that remained unclassified after extensive BLAST based database searching were translated and submitted to OrthoMCL DB and InterProScan. The pattern is similar between unigenes from each transcriptome indicating equivalent unigene classification for *Triphysaria* grown on both hosts. The number of unigenes for which an ortholog or peptide motif was identified was relatively small indicating our unigene classification using PlantTribes 2.0 and external database queries was robust. Approximately 25% of the known orthologs identified in the OrthoMCL database from each transcriptome are shared. A majority of the unigenes remain unknown, and these include many (~500 in each transcriptome) that are >300 nucleotide bp and have read support.

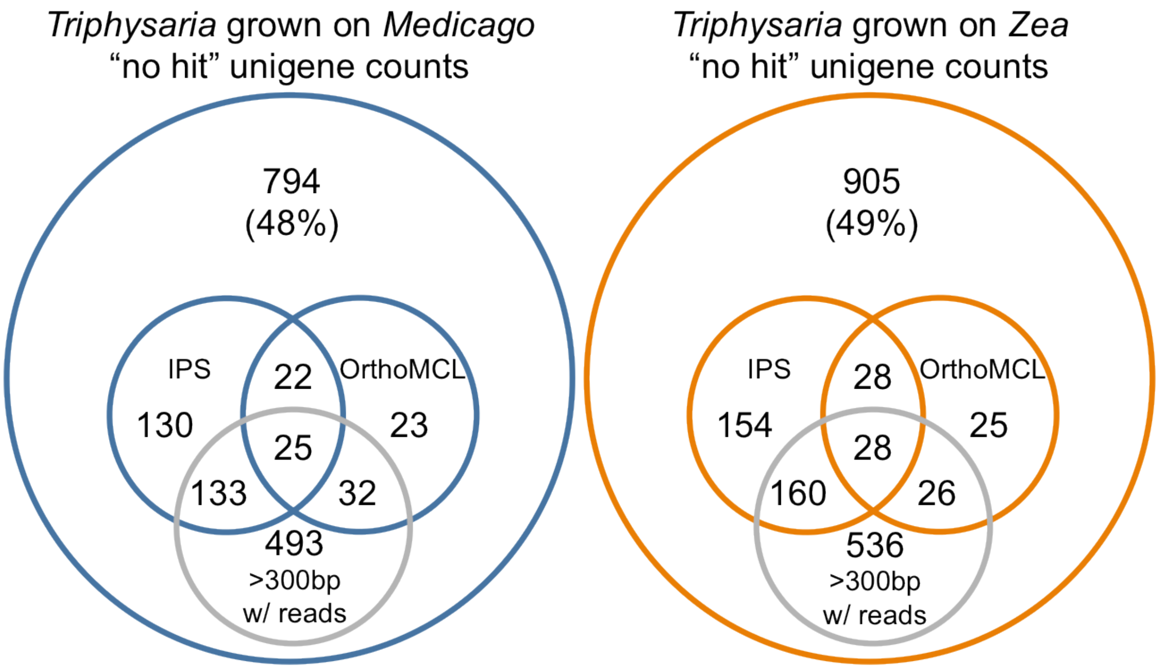

Supplement: Additional file 2: Figure S2 — VENN diagram summary of OrthoMCL DB and InterProScan (IPS) results. ESTScan ORF predictions from unigenes in each interface transcriptome that remained unclassified after extensive BLAST-based database searching were translated and submitted to OrthoMCL DB and InterProScan. The pattern is similar between unigenes from each transcriptome indicating equivalent unigene classification for T. versicolor grown on both hosts. The number of unigenes for which an ortholog or peptide motif was identified was relatively small, indicating our unigene classification using PlantTribes 2.0 and external database queries was robust. Approximately 25% of the known orthologs identified in the OrthoMCL database from each transcriptome are shared.A majority of the unigenes remain unknown, and these include many (~500 in each transcriptome) that are >300 nucleotide bp and have read support. [file 1471-2229-13-9-S2.pdf]
