# Supplementary material for: Functional genomics of a generalist parasitic plant: Laser microdissection of host-parasite interface reveals host-specific patterns of parasite gene expression
Source: BMC Plant Biol. 2013 Jan 9;13:9. doi: 10.1186/1471-2229-13-9 (PMC3636017; doi:10.1186/1471-2229-13-9)
Supplement: Additional file 4: Figure S3 — GO Slim category analysis. Chi-Square test (P<<0.0001) of GO Slim terms represented in the indicated regions of the Venn. The numbers of unigenes in each GO category for indicated regions are listed in the table. Cells with strongly positive residual values (>4) are indicated as bold+ and strongly negative residual values (<-4) are indicated as bold-. GO Slim Function (A), Component (B) and Process (C) category analysis for the interface transcriptome of T. versicolor grown on Z. mays. GO Slim Function (D), Component (E) and Process (F) category analysis for the interface transcriptome of T. versicolor grown on M. truncatula. [file 1471-2229-13-9-S4.pdf]

Supplemental Figure 3A. GO Slim Function category analysis for the interface transcriptome of *T. versicolor* grown on *Z. mays*. Chi-squared test (P=<<0.0001) of GO Slim terms represented in the indicated regions (A-D) of the Venn. The numbers of unigenes in each GO category for regions A-D are indicated in the table. Cells with strongly positive residual values (>4) are indicated as **bold+** and strongly negative residual values (<-4) are indicated as **bold-**.

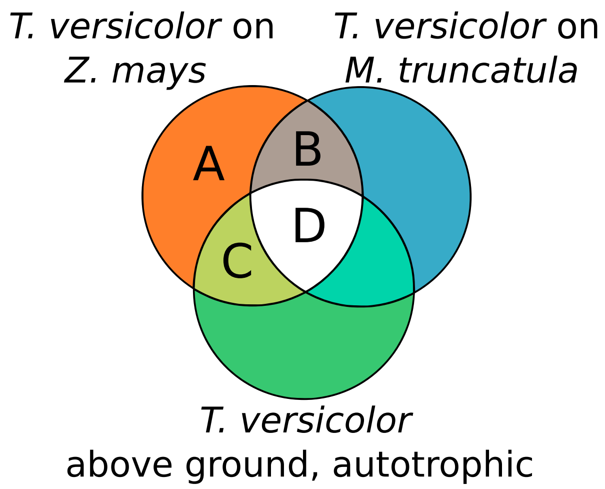

GO Function	A	B	C	D
DNA or RNA binding	24	43	80	515
hydrolase activity	62	177	187	1688
kinase activity	33	70	51	889
nucleic acid binding	3	13	11	152
nucleotide binding	19	**20-**	34	441
other binding	107	205	147	1298
other enzyme activity	66	**146-**	159	1791
other molecular functions	227	**581+**	497	3642
protein binding	32	55	42	644
receptor binding or activity	1	2	0	37
structural molecule activity	**1-**	**12-**	18	**465-**
transcription factor activity	**61+**	**157+**	63	531
transferase activity	**25-**	**78-**	91	1182
transporter activity	21	**66-**	57	974
No GO Function	**123+**	**237+**	121	**768-**

Supplemental Figure 3B. GO Slim Component category analysis for the interface transcriptome of *T. versicolor* grown on *Z. mays*. Chi-squared test (P=<<0.0001) of GO Slim terms represented in the indicated regions (A-D) of the Venn. The numbers of unigenes in each GO category for regions A-D are indicated in the table. Cells with strongly positive residual values (>4) are indicated as **bold+** and strongly negative residual values (<-4) are indicated as **bold-**.

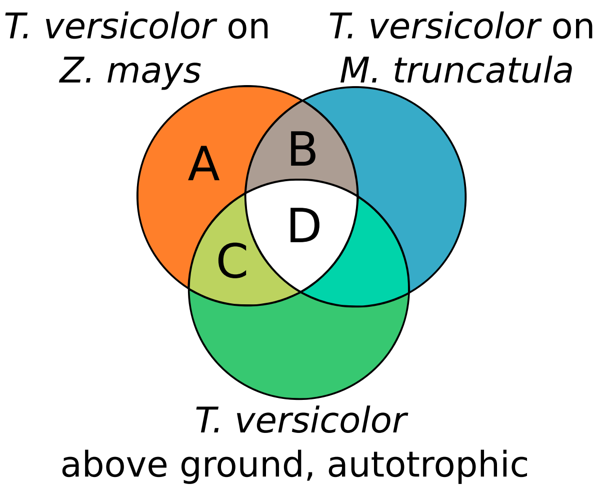

GO Component	A	B	C	D
cell wall	7	8	11	55
chloroplast	59	**110-**	**227+**	1415
cytosol	0	9	12	185
ER	1	22	10	257
extracellular	3	13	3	40
Golgi apparatus	2	12	2	173
mitochondria	18	42	36	647
nucleus	82	189	129	1148
other cellular components	300	656	504	5455
other cytoplasmic components	11	25	31	493
other intracellular components	17	36	24	378
other membranes	150	406	316	3151
plasma membrane	4	10	12	145
plastid	2	5	15	81
ribosome	1	**7-**	17	309
No GO Component	**148+**	**312+**	**209-**	**1085-**

Supplemental Figure 3C. GO Slim Process category analysis for the interface transcriptome of *T. versicolor* grown on *Z. mays*. Chi-squared test (P=<<0.0001) of GO Slim terms represented in the indicated regions (A-D) of the Venn. The numbers of unigenes in each GO category for regions A-D are indicated in the table. Cells with strongly positive residual values (>4) are indicated as **bold+** and strongly negative residual values (<-4) are indicated as **bold-**.

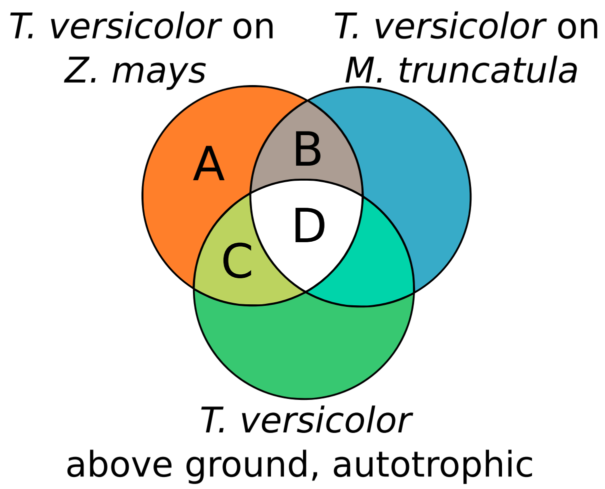

GO Process	A	B	C	D
cell organization and biogenesis	9	27	29	286
developmental processes	14	19	30	145
DNA or RNA metabolism	2	13	5	48
electron transport or energy pathways	21	39	27	298
other biological processes	376	**910+**	711	5827
other cellular processes	47	124	96	1388
other metabolic processes	**147-**	**357-**	398	**4393+**
protein metabolism	1	2	0	14
response to abiotic or biotic stimulus	12	16	28	230
response to stress	2	6	1	65
signal transduction	5	9	15	148
transcription	0	1	3	42
transport	29	**72-**	**58-**	**1157+**
No GO Process	**140+**	**267+**	157	**976-**

Supplemental Figure 3D. GO Slim Function category analysis for the interface transcriptome of *T. versicolor* grown on *M. truncatula*. Chi-squared test (P=<<0.0001) of GO Slim terms represented in the indicated regions (E-H) of the Venn. The numbers of unigenes in each GO category for regions E-H are indicated in the table. Cells with strongly positive residual values (>4) are indicated as **bold+** and strongly negative residual values (<-4) are indicated as **bold-**.

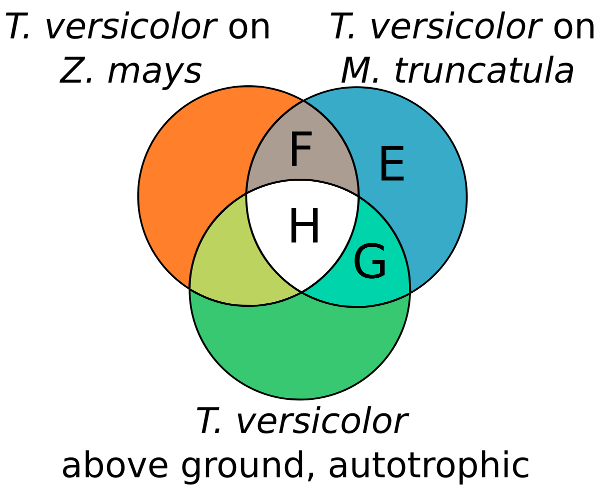

GO Function	E	F	G	H
DNA or RNA binding	9	41	11	444
hydrolase activity	33	156	47	1423
kinase activity	39	66	20	718
nucleic acid binding	2	8	4	118
nucleotide binding	15	22	6	353
other binding	51	184	38	1125
other enzyme activity	20	**132-**	38	1526
other molecular functions	102	**568+**	111	3172
protein binding	12	51	16	569
receptor binding or activity	0	2	0	28
structural molecule activity	2	**10-**	7	446
transcription factor activity	30	**141+**	16	440
transferase activity	25	**64-**	26	991
transporter activity	9	62	11	795
No GO Function	**72+**	**219+**	34	**677-**

Supplemental Figure 3E. GO Slim Component category analysis for the interface transcriptome of *T. versicolor* grown on *M. truncatula*. Chi-squared test (P=<<0.0001) of GO Slim terms represented in the indicated regions (E-H) of the Venn. The numbers of unigenes in each GO category for regions E-H are indicated in the table. Cells with strongly positive residual values (>4) are indicated as **bold+** and strongly negative residual values (<-4) are indicated as **bold-**.

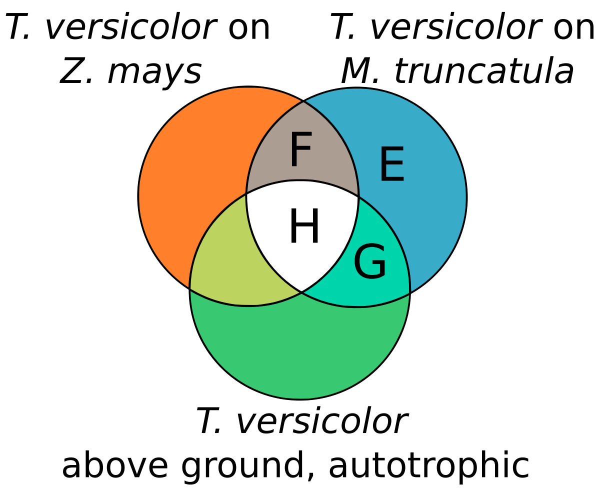

GO Component	E	F	G	H
cell wall	1	6	1	53
chloroplast	20	111	45	1211
cytosol	3	8	2	177
ER	0	15	5	247
extracellular	4	14	1	39
Golgi apparatus	0	12	2	137
mitochondria	0	35	6	544
nucleus	44	169	28	941
other cellular components	152	572	128	4578
other cytoplasmic components	5	29	4	407
other intracellular components	8	36	12	359
other membranes	102	402	86	2657
plasma membrane	0	7	1	120
plastid	2	5	2	75
ribosome	2	**6-**	7	293
No GO Component	**78+**	**299+**	55	**987-**

Supplemental Figure 3F. GO Slim Process category analysis for the interface transcriptome of *T. versicolor* grown on *M. truncatula*. Chi-squared test (P=<<0.0001) of GO Slim terms represented in the indicated regions (E-H) of the Venn. The numbers of unigenes in each GO category for regions E-H are indicated in the table. Cells with strongly positive residual values (>4) are indicated as **bold+** and strongly negative residual values (<-4) are indicated as **bold-**.

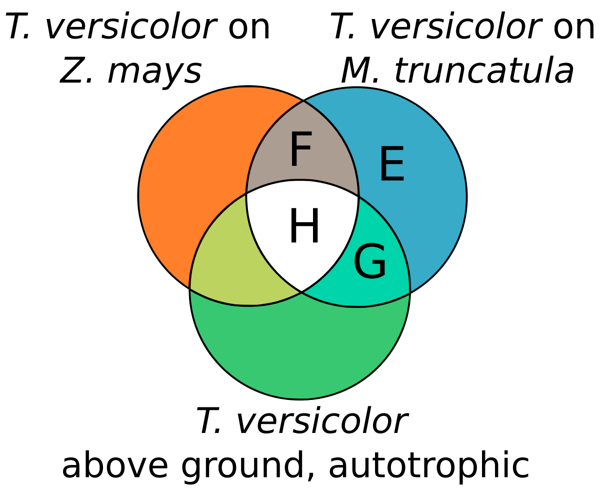

GO Process	E	F	G	H
cell organization and biogenesis	6	25	7	291
developmental processes	4	15	8	130
DNA or RNA metabolism	4	12	2	50
electron transport or energy pathways	10	45	16	237
other biological processes	198	**854+**	177	4946
other cellular processes	53	116	25	1193
other metabolic processes	**62-**	**310-**	89	3750
protein metabolism	0	1	0	12
response to abiotic or biotic stimulus	5	12	3	173
response to stress	0	6	1	59
signal transduction	2	10	2	124
transcription	0	1	0	36
transport	8	**66-**	13	967
No GO Process	**69+**	**253+**	42	**857-**
