# Supplementary material for: Functional genomics of a generalist parasitic plant: Laser microdissection of host-parasite interface reveals host-specific patterns of parasite gene expression
Source: BMC Plant Biol. 2013 Jan 9;13:9. doi: 10.1186/1471-2229-13-9 (PMC3636017; doi:10.1186/1471-2229-13-9)

Supplemental Figure 4. Correlation of normalized read counts (RPKM) for unigenes in orthogroups shared between the interface transcriptomes and reference assembly TrVeBC1 (ppgp.huck.psu.edu). Reads from each interface transcriptome were mapped to a reference assembly (TrVeBC2, ppgp.huck.psu.edu) that included whole haustorium data from *T. versicolor* grown on *M. truncatula*. There are a subset of unigenes that are more highly expressed in the interface transcriptome of *T. versicolor* grown on *M. truncatula*, yet not a similar pattern for the *T. versicolor* grown on *Z. mays*. This is due to a bias for *Medicago* grown *Triphysaria* unigenes in the reference dataset TrVeBC2, which was constructed with reads from *Medicago* grown *Triphysaria*. For unigenes in shared orthogroups, the RPKM values are highly correlated (Pearson's R = 0.81) between interface transcriptomes indicating that technical and biological variation is low.

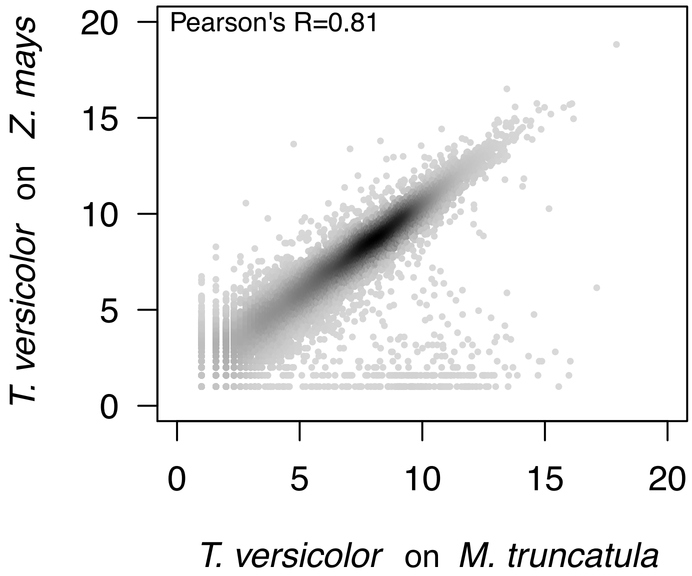

Supplement: Additional file 5: Figure S4 — Correlation of normalized read counts (RPKM) for unigenes in orthogroups shared between the interface transcriptomes and reference assembly TrVeBC1 (ppgp.huck.psu.edu). Reads from each interface transcriptome were mapped to a reference assembly (TrVeBC2, ppgp.huck.psu.edu) that included whole haustorium data from T. versicolor grown on M. truncatula. A subset of unigenes is more highly expressed in the interface transcriptome of T. versicolor grown on M. truncatula; a similar pattern is not observed for T. versicolor grown on Z. mays. This is due to a bias for Medicago grown Triphysaria unigenes in the reference dataset TrVeBC2, which was constructed with reads from Medicago grown Triphysaria. For unigenes in shared orthogroups, the RPKM values are highly correlated (Pearson’s R = 0.81) between interface transcriptomes indicating that technical and biological variation is low. [file 1471-2229-13-9-S5.pdf]
