# Supplementary material for: Functional genomics of a generalist parasitic plant: Laser microdissection of host-parasite interface reveals host-specific patterns of parasite gene expression
Source: BMC Plant Biol. 2013 Jan 9;13:9. doi: 10.1186/1471-2229-13-9 (PMC3636017; doi:10.1186/1471-2229-13-9)
Supplement: Additional file 6: Figure S5 — Highly Expressed Interface Unigenes. The 20 most highly expressed (RPKM) unigenes (ID) in each indicated portion of the transcriptome Venn diagram for the interaction of T. versicolor with each host species. NR BLASTx – description, species and %id.: the description, species of origin, and percent pairwise identify, respectively, of the best unigene alignment (<1e-10) resulting from the NR database query, 17 genomes BLAST and %id.: best hit species in a BLAST database of 17 annotated plant genomes with the percent pairwise identity in the nucleotide BLAST (N) or translated nucleotide BLAST (P). TXXX = IPS transmembrane prediction, SXXX = IPS secretion signal prediction. [file 1471-2229-13-9-S6.pdf]

Supplemental Figure 5. Highly Expressed Interface Unigenes. The 20 most highly expressed (**RPKM**) unigenes (**ID**) in each indicated portion of the transcriptome Venn diagram for the interaction of *T. versicolor* with each host species. **NR BLASTx – description, species** and **%id.**: the description, species of origin, and percent pairwise identify, respectively, of the best unigene alignment (>1e-10) resulting from the NR database query, **17 genomes BLAST** and **%id.:** best hit species in a BLAST database of 17 annotated plant genomes with the percent pairwise identity in the nucleotide BLAST (**N**) or translated nucleotide BLAST (**P**). ^{T}XXX = IPS transmembrane prediction, ^{S}XXX = IPS secretion signal prediction.

Supplemental Fig. 5A. Top expressed unigenes from shared orthogroups between parasite-host interface transcriptomes of *T. versicolor.*

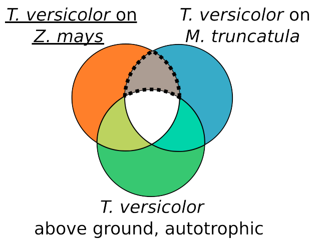

RPKM	ID	NR BLASTx – description	NR BLASTx species	% id.	17 genomes BLAST	% id.
4351	772	[TS]beta-expansin precursor	*Vitis vinifera*	67	*Mimulus guttatus*	57 P
2411	2150	disease resistance-responsive (dirigent-like)	*Populus trichocarpa*	78	*Populus trichocarpa*	63 P
1464	1122	[S]cysteine-rich repeat secretory protein 38	*Arabidopsis thaliana*	56	*Mimulus guttatus*	49 P
1277	11517	[TS]PREDICTED: uncharacterized protein	*Glycine max*	90	*Solanum tuberosum*	72 P
972	3187	pathogenesis-related protein sth-2	*Vitis vinifera*	69	*Vitis vinifera*	45 P
836	571	PREDICTED: At5g39570-like	*Glycine max*	54	*Solanum lycopersicum*	52 P
733	11012	[T]nodule protein dg93-like protein	*Potamogeton distinctus*	90	*Mimulus guttatus*	71 P
631	15459	pathogenesis-related protein	*Rehmannia glutinosa*	70	*Mimulus guttatus*	47 P
616	494	acidic endochitinase	*Populus trichocarpa*	71	*Populus trichocarpa*	57 P
560	5467	[TS]rsi-1 protein	*Solanum lycopersicum*	82	*Populus trichocarpa*	81 N
493	3586	ap2 erf domain-containing transcription factor	*Populus trichocarpa*	62	*Mimulus guttatus*	89 N
453	16555	[S]PREDICTED: chemocyanin-like	*Brachypodium distachyon*	54	*Theobroma cacao*	43 P
448	19049	PREDICTED: putative expansin-B2	*Vitis vinifera*	72	*Mimulus guttatus*	54 P
412	7820	major allergen pru ar 1	*Vitis vinifera*	79	*Mimulus guttatus*	71P
399	2405	Pectinesterase-1 precursor, putative	*Ricinus communis*	83	*Mimulus guttatus*	82 P
397	6864	cysteine-rich repeat secretory protein 38-like	*Vitis vinifera*	60	*Glycine max*	47 P
380	7395	pathogenesis-related protein 1a	*Vitis vinifera*	69	*Vitis vinifera*	52 P
379	259	[TS]strictosidine synthase 1-like	*Vitis vinifera*	66	*Vitis vinifera*	49 P
374	328	[S]peroxidase protein	*Mikania micrantha*	86	*Solanum tuberosum*	74 N
369	193	[S]cytokinin dehydrogenase 3- partial	*Vitis vinifera*	66	*Mimulus guttatus*	59 P

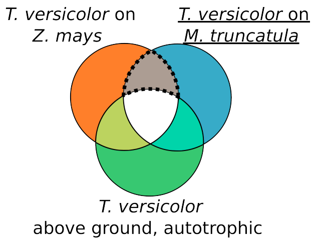

RPKM	ID	NR BLASTx – description	NR BLASTx species	% id.	17 genomes BLAST	% id.
19043	12004	pathogenesis-related protein	*Rehmannia glutinosa*	69	*Mimulus guttatus*	50 P
7238	2255	pathogenesis-related protein	*Rehmannia glutinosa*	69	*Mimulus guttatus*	48 P
6910	6809	pathogenesis-related protein sth-2	*Vitis vinifera*	68	*Vitis vinifera*	44 P
4107	12313	pathogenesis-related protein	*Rehmannia glutinosa*	70	*Medicago truncatula*	48 P
3525	331	pathogen-related protein sth-2	*Salvia miltiorrhiza*	80	*Mimulus guttatus*	71 P
2151	11591	pathogenesis-related protein	*Rehmannia glutinosa*	71	*Mimulus guttatus*	50 P
1396	416	[TS]cysteine-rich repeat secretory protein	*Vitis vinifera*	55	*Solanum tuberosum*	42 P
1187	7825	[TS]chemocyanin precursor	*Populus trichocarpa*	65	*Populus trichocarpa*	57 P
1138	20761	pathogenesis-related protein	*Rehmannia glutinosa*	68	*Mimulus guttatus*	50 P
1132	11358	[TS]PREDICTED: uncharacterized protein	*Glycine max*	89	*Solanum tuberosum*	73 P
1086	6	[S]aspartic proteinase nepenthesin-1	*Ricinus communis*	54	*Mimulus guttatus*	83 P
1045	5067	[TS]NA	NA	NA	*Sorghum bicolor*	40 P
1029	4931	[TS]chemocyanin precursor	*Populus trichocarpa*	54	*Populus trichocarpa*	37 P
906	837	[S]cysteine-rich repeat secretory protein 38	*Arabidopsis thaliana*	51	*Arabidopsis thaliana*	36 P
900	2050	[S]disease resistance response protein 206-like	*Vitis vinifera*	69	*Vitis vinifera*	53 P
707	1010	cysteine-rich repeat secretory protein 38	*Arabidopsis thaliana*	56	*Mimulus guttatus*	49 P
652	13435	hypothetical protein ARALYDRAFT_473065	*Arabidopsis lyrata*	73	*Mimulus guttatus*	63 P
620	7456	predicted protein	*Populus trichocarpa*	62	*Populus trichocarpa*	50 P
**574**	**5537**	**pathogenesis-related protein 1a**	***Medicago truncatula***	**97**	***Medicago truncatula***	**94 N**
566	5883	[T]predicted protein	*Populus trichocarpa*	80	*Populus trichocarpa*	70 P

Supplemental Figure 5B. Top expressed unigenes from orthogroups unique to parasite-host interface transcriptomes *T. versicolor.*

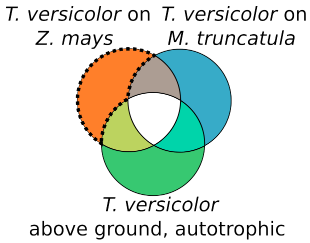

RPKM	ID	NR BLASTx – description	NR BLAST species	% id.	17 genomes BLAST	% id.
1711	9854	[S]NA	NA	NA	*Theobroma cacao*	40 P
551	611	[TS]zinc transporter	*Nicotiana tabacum*	79	*Mimulus guttatus*	76 P
416	3088	predicted protein	*Populus trichocarpa*	97	*Mimulus guttatus*	96 P
409	11956	protein kti12 homolog	*Populus trichocarpa*	92	*Vitis vinifera*	88 N
313	4049	hypothetical protein SORBIDRAFT_06g029810	*Sorghum bicolor*	92	*Sorghum bicolor*	89 N
281	710	[T]zinc transporter 5 isoform 1	*Vitis vinifera*	80	*Mimulus guttatus*	81 P
279	9521	NA	NA	NA	*Solanum tuberosum*	57 P
190	1258	[TS]zinc iron	*Ricinus communis*	86	*Mimulus guttatus*	81 N
162	1590	polygalacturonase pectinase	*Arabidopsis lyrata*	77	*Mimulus guttatus*	76 P
152	14303	gdsl esterase lipase at3g62280-like	*Vitis vinifera*	62	*Solanum lycopersicum*	45 P
146	12610	conserved hypothetical protein	*Ricinus communis*	54	*Mimulus guttatus*	56 P
136	4574	PREDICTED: uncharacterized protein LOC100254926	*Vitis vinifera*	86	*Mimulus guttatus*	88 P
101	14713	[S]uncharacterized wd repeat-containing protein alr3466-	*Vitis vinifera*	80	*Solanum tuberosum*	68 P
101	13325	dna-binding protein escarola-like	*Vitis vinifera*	55	*Vitis vinifera*	46 P
100	7730	af302808_1metallothionein-like protein	*Sesamum indicum*	84	*Mimulus guttatus*	70 P
99	14437	proteinase inhibitor	*Jatropha curcas*	87	*Mimulus guttatus*	73 P
97	9225	[TS]peptide nitrate transporter	*Populus trichocarpa*	80	*Populus trichocarpa*	66 P
96	5684	NA	NA	NA	*Mimulus guttatus*	47 P
95	5440	[T]NA	NA	NA	*Populus trichocarpa*	36 P
**94**	**14819**	**NA**	**NA**	**NA**	***Sorghum bicolor***	**94 N**

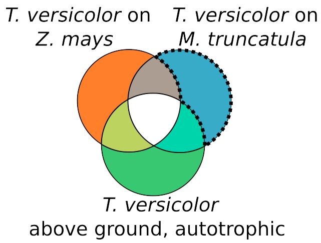

667	2072	[S]brassinosteroid insensitive 1-associated receptor kinase	*Vitis vinifera*	60	*Vitis vinifera*	47 P
419	4028	lob domain-containing protein 41-like	*Glycine max*	52	*Mimulus guttatus*	64 P
344	337	PREDICTED: uncharacterized protein LOC100788950	*Glycine max*	68	*Carica papaya*	53 P
148	15159	[TS] proteinase inhibitor	*Oryza sativa Japonica*	69	*Fragaria vesca*	55 P
146	2274	PREDICTED: uncharacterized protein LOC100793448	*Glycine max*	82	*Mimulus guttatus*	70 P
145	6381	NA	NA	NA	*Glycine max*	43 P
121	376	[S]isoliquiritigenin 2 -o-methyltransferase-like	*Glycine max*	82	*Glycine max*	78 N
111	1550	[TS]casp-like protein 9-like	*Glycine max*	93	*Glycine max*	86 N
98	5544	zinc finger	*Populus trichocarpa*	84	*Mimulus guttatus*	81 P
97	8801	PREDICTED: uncharacterized protein LOC100854976	*Vitis vinifera*	72	*Mimulus guttatus*	64 P
95	4555	[T]disease resistance protein	*Glycine max*	79	*Glycine max*	73 P
95	646	PREDICTED: uncharacterized protein LOC100776135	*Glycine max*	67	*Mimulus guttatus*	79 N
92	5876	PREDICTED: uncharacterized protein LOC100258555	*Vitis vinifera*	77	*Carica papaya*	80 N
90	3020	ribosomal protein l16	*Nicotiana tabacum*	98	*Solanum lycopersicum*	96 N
81	9746	predicted protein	*Populus trichocarpa*	81	*Mimulus guttatus*	75 P
79	15676	predicted protein	*Populus trichocarpa*	95	*Mimulus guttatus*	82 N
**77**	**6390**	**germin-like protein**	***Medicago truncatula***	**100**	***Medicago truncatula***	**94 N**
72	1213	embryonic element binding factor 7	*Daucus carota*	73	*Solanum lycopersicum*	84 N
72	11016	chaperone protein	*Ricinus communis*	72	*Mimulus guttatus*	77 N
70	6242	dna binding	*Ricinus communis*	77	*Theobroma cacao*	60 P

Supplemental Figure 5C. Top expressed unigenes from host-specific yet above ground-shared orthogroups of *T. versicolor transcriptomes.*

T. versicolor on *Z. mays* — *T. versicolor* on *M. truncatula*

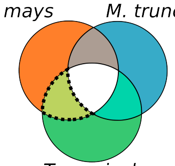

T. versicolor above ground, autotrophic

RPKM	ID	NR BLASTx – description	NR BLAST species	% id.	17 genomes BLAST	% id.
919	6408	protein auxin response 4	*Vitis vinifera*	66	*Fragaria vesca*	56 P
834	463	conserved hypothetical protein	*Ricinus communis*	79	*Mimulus guttatus*	78 N
766	2862	[TS]disease resistance response	*Vitis vinifera*	68	*Solanum tuberosum*	57 P
523	277	atp-citrate synthase beta chain protein 2	*Populus trichocarpa*	95	*Mimulus guttatus*	88 N
453	7624	UP-9A	*Nicotiana tabacum*	86	*Mimulus guttatus*	85 N
329	59	PREDICTED: uncharacterized protein isoform 1	*Glycine max*	75	*Solanum tuberosum*	78 N
274	4716	armadillo beta-catenin-like repeat-containing protein	*Glycine max*	91	*Solanum lycopersicum*	79 N
266	10508	seed maturation protein pm35	*Phaseolus vulgaris*	77	*Solanum tuberosum*	69 P
265	12720	[TS]hydrophobic protein	*Ricinus communis*	93	*Mimulus guttatus*	74 P
240	14096	betanidin-5-O-glucosyltransferase	*Dorotheanthus bellidiformis*	72	*Solanum tuberosum*	64 P
232	14660	conserved hypothetical protein	*Ricinus communis*	80	*Mimulus guttatus*	68 P
231	3754	PREDICTED: uncharacterized protein LOC100263294	*Vitis vinifera*	53	*Mimulus guttatus*	43 P
229	10547	NA	NA	NA	*Mimulus guttatus*	59 P
218	512	PREDICTED: uncharacterized protein LOC100252003	*Vitis vinifera*	62	*Mimulus guttatus*	71 P
206	1803	phosphate abc	*Ricinus communis*	91	*Solanum tuberosum*	82 P
184	2144	fact complex subunit spt16	*Medicago truncatula*	90	*Phoenix dactylifera*	76 P
177	12962	auxin-induced cp12 domain-containing protein	*Pisum sativum*	94	*Medicago truncatula*	82 P
171	10358	NA	NA	NA	*Mimulus guttatus*	90 N
171	10892	[T]hypothetical protein	*Citrus unshiu*	80	*Mimulus guttatus*	72 P
164	8348	kidney mitochondrial carrier protein 1-like	*Vitis vinifera*	96	*Mimulus guttatus*	98 P

T. versicolor on *Z. mays* — *T. versicolor* on *M. truncatula*

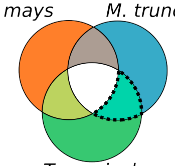

T. versicolor above ground, autotrophic

RPKM	ID	NR BLASTx – description	NR BLAST species	% id.	17 genomes BLAST	% id.
2772	24566	PREDICTED: uncharacterized protein LOC100801029	*Glycine max*	78	*Carica papaya*	89 N
263	**147**	[TS]**aquaporin pip2-7**	***Medicago truncatula***	98	***Medicago truncatula***	**94 N**
249	9620	ubiquitin-fold modifier 1-like	*Glycine max*	100	*Mimulus guttatus*	89 N
199	16638	small blue copper protein bcp1	*Paraboea crassifolia*	75	*Mimulus guttatus*	70 P
181	1393	[T]peroxisomal membrane protein	*Ricinus communis*	89	*Mimulus guttatus*	84 N
169	22366	PREDICTED: uncharacterized protein At4g14450	*Glycine max*	59	*Mimulus guttatus*	70 P
159	84	[S]fructokinase 2	*Petunia integrifolia subsp.*	92	*Mimulus guttatus*	81 N
125	10027	[S]predicted protein	*Populus trichocarpa*	90	*Phoenix dactylifera*	77 N
124	1041	inactive poly	*Ricinus communis*	65	*Mimulus guttatus*	61 P
119	7997	small nuclear ribonucleoprotein e	*Glycine max*	98	*Carica papaya*	97 P
104	10651	candidate beta-glucosidase, glycoside hydrolase family 1	*Postia placenta Mad-698-R*	82	*Mimulus guttatus*	82 P
103	107	hypothetical protein	*Plantago major*	55	*Mimulus guttatus*	46 P
97	1307	macro domain-containing protein vpa0103	*Vitis vinifera*	90	*Mimulus guttatus*	84 P
91	8780	[TS]lysine histidine transporter	*Plantago major*	96	*Carica papaya*	91 P
88	3129	PREDICTED: uncharacterized protein	*Vitis vinifera*	94	*Mimulus guttatus*	63 P
87	9545	regulatory-associated protein of tor 1-like isoform 1	*Vitis vinifera*	88	*Mimulus guttatus*	90 P
83	2665	cytochrome p450 81d1-like	*Glycine max*	85	*Fragaria vesca*	80 N
80	12099	[S]PREDICTED: uncharacterized protein	*Glycine max*	58	*Mimulus guttatus*	88 P
75	7457	[T]uncharacterized gpi-anchored protein at4g28100-like	*Vitis vinifera*	73	*Mimulus guttatus*	64 P
74	5779	atp binding	*Ricinus communis*	70	*Solanum lycopersicum*	57 P
