# Supplementary material for: Functional genomics of a generalist parasitic plant: Laser microdissection of host-parasite interface reveals host-specific patterns of parasite gene expression
Source: BMC Plant Biol. 2013 Jan 9;13:9. doi: 10.1186/1471-2229-13-9 (PMC3636017; doi:10.1186/1471-2229-13-9)
Supplement: Additional file 11: Figure S6 — RaxML analysis of A: Triphysaria beta expansin gene TvEXPB1 (TrVeIntZeamaGB1_772, green text), and B: alpha expansin gene TvEXPA4 (TrVeIntMedtrGB1_11, green text). Bootstrap proportions are given above each node. Taxon abbreviations for A: Arabidopsis thaliana (AT), Oryza sativa (Os), Mimulus guttatus (Mg), Triphysaria versicolor (TrVe), Striga hermonthica (StHe), Phelipanche (=Orobanche) aegyptiaca (OrAe), Selaginella moellendorffii (Smoellendorffii). Taxon Abbreviations for B:Oryza sativa (Os), Sorghum bicolor (Sb), Striga hermonthica (StHe), Phelipanche (=Orobanche) aegyptiaca (OrAe), Triphysaria versicolor (TrVe), Carica papaya (Carpa), Populus trichocarpa (Poptr), Medicago truncatula (Medtr), Vitis vinifera (Vitvi), Arabidopsis thaliana (AT), Selaginella moellendorffii (Selmo), Physcomitrella patens (Phypa). [file 1471-2229-13-9-S11.pdf]

Supplemental Figure 6. RaxML analysis of A: *Triphysaria* beta expansin gene TvEXPB1 (TrVeIntZeamaGB1_772*, green text), and B: alpha expansin gene TvEXPA4 (TrVeIntMedtrGB1_11*, green text). Bootstrap proportions are given above each node. **Taxon abbreviations for A**: *Arabidopsis thaliana* (AT), *Oryza sativa* (Os), *Mimulus guttatus* (Mg), *Triphysaria versicolor* (TrVe), *Striga hermonthica* (StHe), *Phelipanche* (=*Orobanche*) *aegyptiaca* (OrAe), *Selaginella mollendorfii* (Smollendorfii). **Taxon Abbreviations for B**: *Oryza sativa* (Os), *Sorghum bicolor* (Sb), *Striga hermonthica* (StHe), *Phelipanche* (=*Orobanche*) *aegyptiaca* (OrAe), *Triphysaria versicolor* (TrVe), *Carica papaya* (Carpa), *Populus trichocarpa* (Poptr), *Medicago truncatula* (Medtr), *Vitis vinifera* (Vitvi), *Arabidopsis thaliana* (AT), *Selaginella mollendorfii* (Selmo), *Physcomitrella patens* (Phypa).

A

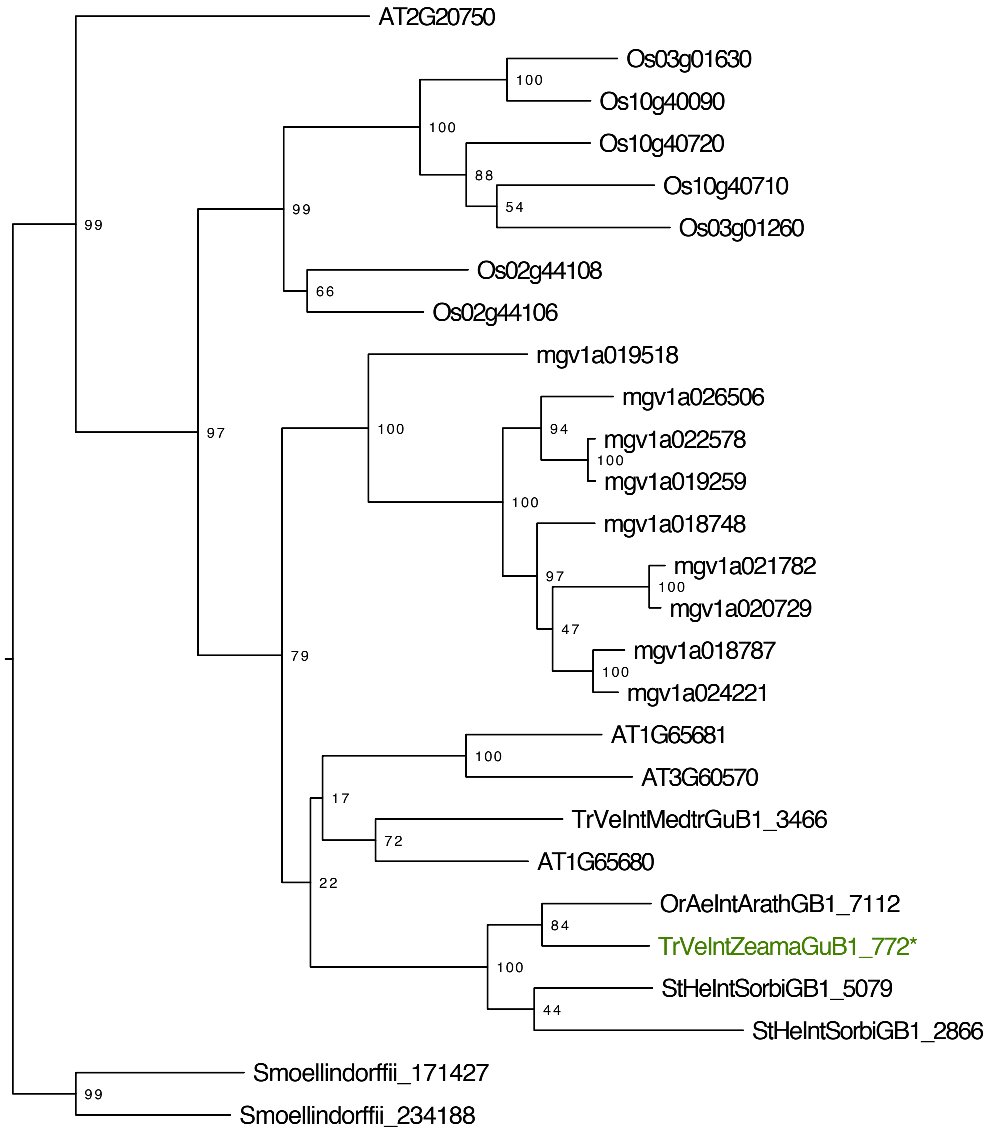

B

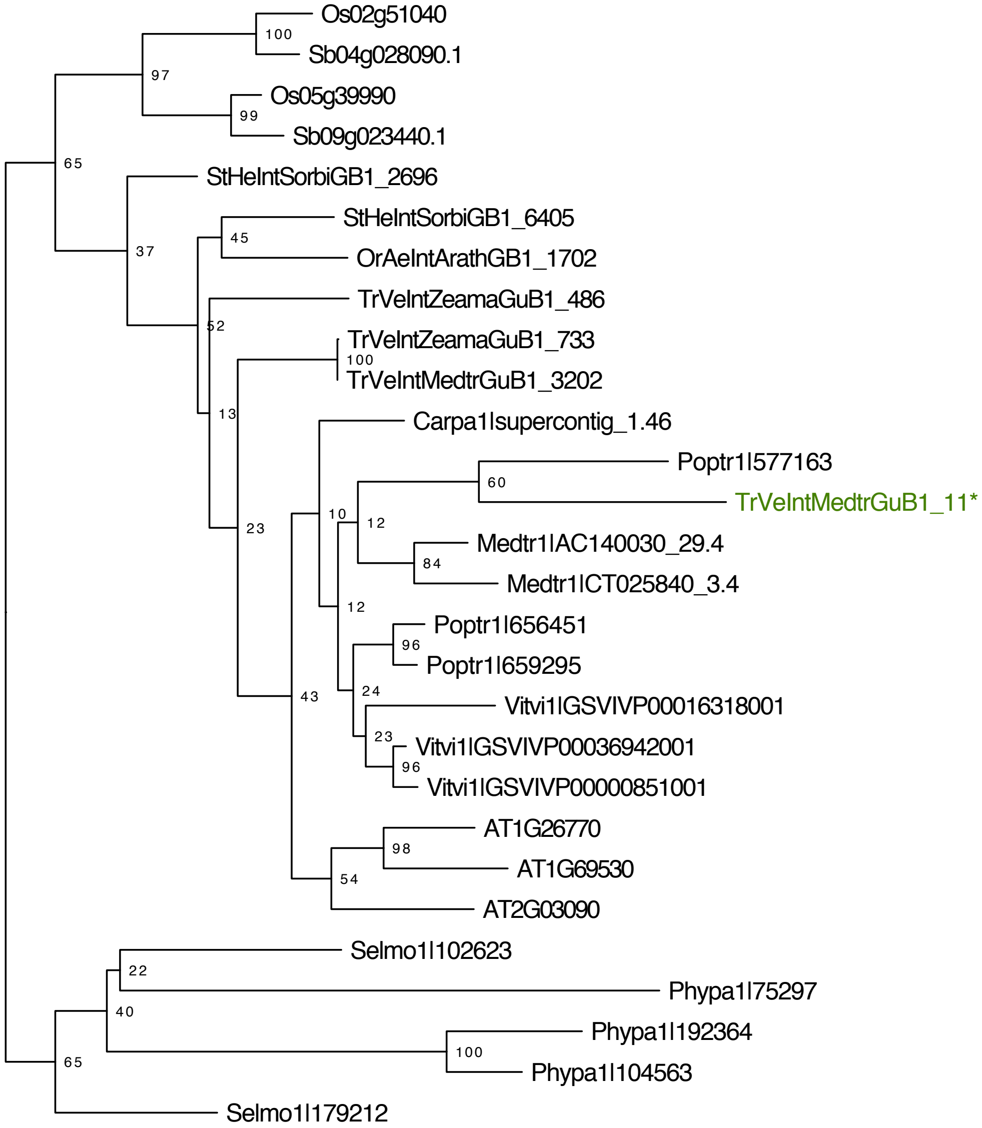

Os02g51040
100
Sb04g028090.1
97
Os05g39990
99
Sb09g023440.1
65
StHeIntSorbiGB1_2696
StHeIntSorbiGB1_6405
45
OrAeIntArathGB1_1702
37
TrVeIntZeamaGuB1_486
52
TrVeIntZeamaGuB1_733
100
TrVeIntMedtrGuB1_3202
13
Carpa1lsupercontig_1.46
Poptr1l577163
60
TrVeIntMedtrGuB1_11*
10
12
Medtr1lAC140030_29.4
84
Medtr1lCT025840_3.4
23
12
Poptr1l656451
96
Poptr1l659295
24
Vitvi1lGSVIVP00016318001
23
Vitvi1lGSVIVP00036942001
96
Vitvi1lGSVIVP00000851001
43
AT1G26770
98
AT1G69530
54
AT2G03090
Selmo1l102623
22
Phypa1l75297
40
Phypa1l192364
100
Phypa1l104563
65
Selmo1l179212
